# Engaging the private sector in malaria surveillance: a review of strategies and recommendations for elimination settings

**DOI:** 10.1186/s12936-017-1901-1

**Published:** 2017-06-14

**Authors:** Adam Bennett, Anton L. V. Avanceña, Jennifer Wegbreit, Chris Cotter, Kathryn Roberts, Roly Gosling

**Affiliations:** 1 Malaria Elimination Initiative, UCSF Global Health Group, 550 16th Street, 3rd Floor, San Francisco, CA 94158 USA; 2 Department of Epidemiology & Biostatistics, School of Medicine, University of California, San Francisco, 550 16th Street, 2nd Floor, San Francisco, CA 94158 USA

**Keywords:** Malaria, Malaria elimination, Malaria surveillance, Private sector, Private sector engagement, Swaziland, Vietnam

## Abstract

**Background:**

In malaria elimination settings, all malaria cases must be identified, documented and investigated. To facilitate complete and timely reporting of all malaria cases and effective case management and follow-up, engagement with private providers is essential, particularly in settings where the private sector is a major source of healthcare. However, research on the role and performance of the private sector in malaria diagnosis, case management and reporting in malaria elimination settings is limited. Moreover, the most effective strategies for private sector engagement in malaria elimination settings remain unclear.

**Methods:**

Twenty-five experts in malaria elimination, disease surveillance and private sector engagement were purposively sampled and interviewed. An extensive review of grey and peer-reviewed literature on private sector testing, treatment, and reporting for malaria was performed. Additional in-depth literature review was conducted for six case studies on eliminating and neighbouring countries in Southeast Asia and Southern Africa.

**Results:**

The private health sector can be categorized based on their commercial orientation or business model (for-profit versus nonprofit) and their regulation status within a country (formal *vs* informal). A number of potentially effective strategies exist for engaging the private sector. Conducting a baseline assessment of the private sector is critical to understanding its composition, size, geographical distribution and quality of services provided. Facilitating reporting, referral and training linkages between the public and private sectors and making malaria a notifiable disease are important strategies to improve private sector involvement in malaria surveillance. Financial incentives for uptake of rapid diagnostic tests and artemisinin-based combination therapy should be combined with training and community awareness campaigns for improving uptake. Private sector providers can also be organized and better engaged through social franchising, effective regulation, professional organizations and government outreach.

**Conclusion:**

This review highlights the importance of engaging private sector stakeholders early and often in the development of malaria elimination strategies.

**Electronic supplementary material:**

The online version of this article (doi:10.1186/s12936-017-1901-1) contains supplementary material, which is available to authorized users.

## Background

The private sector is often the first source of primary healthcare services, particularly for children [1–9]. These patterns are similar for malaria treatment, and in some regions up to three-quarters of all treatment-seeking for fevers occur in the private health sector [10–16]. The prominent role of the private health sector for healthcare services is likely a result of the greater availability and ease of access to private providers, greater flexibility in prescribing medicine, greater availability of anti-malarials (although often not frontline drugs) and perceptions of the relative quality of services compared to the public sector [17–24]. The rural poor, who are often at higher risk of malaria infection, are also more likely to use informal private providers in many settings [25, 26].

Given its diversity and reach, the private health sector is an essential partner for malaria surveillance and represents an underutilized resource for malaria programmes to deliver effective healthcare to populations with limited access to the public sector [27–33]. For example, the private sector may be best suited to provide case management for mobile and hard-to-reach populations, as private providers may be more conveniently located in high-risk communities or in border areas. However, knowledge on the most effective approaches to ensure availability and appropriate use of diagnostics and treatment and complete reporting of malaria cases is limited.

Malaria surveillance—which includes testing, treatment and reporting of malaria cases—in countries approaching malaria elimination differs in some key ways compared to high-burden settings [34]. In high-burden, control settings, surveillance staff often only have the resources to report aggregate case numbers, and the focus of case management is primarily on preventing severe disease and death [35]. The availability and appropriate use of diagnostics is important for ensuring that case counts reflect confirmed malaria cases. In low-transmission and elimination settings, malaria infections tend to be more clustered in space and time, and the surveillance system needs to be more sensitive to detect these more dispersed transmission foci. Further, in elimination settings, all cases and potential foci of transmission need to be investigated in a timely manner in order to prevent onward transmission of the disease [35–40]. Certification of malaria elimination status also requires a detailed documentation of all cases and foci during this final phase, and as a result, relies upon complete, robust participation of the entire health system.

Engaging the private health sector in malaria surveillance is essential for reducing malaria transmission, especially in settings where the private sector is a major source of healthcare. However, research on the most effective strategies to do so remains limited. This paper seeks to fill this gap by synthesizing research and expert knowledge on the current state of the private health sector’s role in malaria surveillance, including common challenges faced by malaria programmes in engaging the private sector. Drawing on broad experiences from various transmission settings, this paper reviews effective strategies for improving private sector testing, treatment, tracking and reporting of malaria cases, with a focus on malaria elimination contexts.

## Methods

This paper utilized qualitative methods and was informed by interviews and e-mail correspondences with key informants and an extensive review of grey and published literature.

A purposive sample of key informants was used. First, a list of domain experts working on malaria elimination programmes or private sector engagement was identified. These experts were then contacted via e-mail and invited for in-depth, semi-structured interviews in person, over the phone or through video teleconferencing. Interview questions were open-ended and focused on private sector diagnosis, treatment and reporting of malaria cases. Key informants were asked to comment on the role of the private sector in malaria surveillance globally, and specifically in countries and regions where they had direct experience. Additional key informants were identified and interviewed based on the endorsements and recommendations of the initial key informants. Qualitative data were organized in Microsoft Excel^®^ based on predetermined themes that were framed by the interview guide (Table 1).

**Table 1:** Key informant questions

Theme	Question
Definition and composition of private sector in the context of malaria surveillance	What exactly does “private sector” mean when talking about malaria surveillance? What types of private sector entities have been involved or should be involved in malaria surveillance?
Ideal versus current private sector involvement in malaria surveillance	Thinking about the private sector entities you just mentioned, ideally, how do you think they should be involved in malaria surveillance? How does this “ideal involvement” compare to how things currently function?
Challenges or issues with private sector engagement	What are some of the key challenges for malaria programmes when engaging the private sector for malaria surveillance? Where possible, please comment on specific countries that have attempted to engage the private sector. What are some of the key challenges they have experienced? How were these addressed?
Models or examples of private sector engagement	What countries or surveillance systems have successfully engaged the private sector? Can you tell us about the ones that have been most successful?
Incentives and regulations for the private sector	What do you think are the key steps to improving involvement of the private sector in malaria surveillance?
Promising approaches in private sector engagement	What ongoing research, programmes, or initiatives exist to engage the private sector in malaria surveillance that you think is particularly promising?
Gaps in private sector research	If you had the opportunity to design either research or a pilot project to investigate a private sector engagement strategy for malaria elimination, what would you do?
Examples from other diseases or health programmes	Thinking about diseases outside of malaria, are there any examples of how the private sector has been successfully engaged that could be transferred to the malaria sphere?

Literature reviews were conducted for two purposes. First, published and grey literature on the private health sector in relation to malaria surveillance was sought and collated. The private health sector was defined as including any non-state actor or provider of healthcare services. Second, relevant articles, studies and reports were gathered for six country case studies—three in Southeast Asia (Cambodia, Myanmar and Vietnam) and three in Southern Africa (Mozambique, Swaziland and Zambia). These countries represent a mix of malaria-eliminating and neighboring countries with sub-national elimination goals, and countries that belong to regional networks with stated malaria elimination goals [41–44]. These countries were also chosen because they have employed different private sector engagement strategies that may have regional implications. Challenges and lessons from these case studies were used to make broad recommendations.

For the literature reviews, MEDLINE (via PubMed) and SCOPUS were searched for relevant peer-reviewed articles in English published on or before May 31, 2015. Between October 2014 and May 2015, recurring online searches for grey literature, reports, policy documents and web articles were also conducted. For both searches, “disease,” “malaria,” “malaria elimination” and “private sector” were combined with one or more of following search terms: “public–private partnerships,” “public health surveillance,” “diagnosis,” “therapeutics,” “testing,” “treatment,” “disease notification,” “disease reporting,” “engagement,” “strategies,” involvement,” “model,” and “examples”. For the country case studies, the countries’ names were included in the searches. Multiple key informants also sent unpublished reports or documents to which they had access.

Articles that did not relate to private sector surveillance based on their titles and abstracts were excluded. Articles that were found to be relevant based on this initial screening were assessed further for relevance through a full-text review. Articles were excluded at this stage if the information they presented was not related to malaria surveillance (e.g., vaccines) or the private sector’s role in malaria surveillance. The final list of included articles was organized by topic or theme.

### Ethics statement

Ethical approval was sought from the University of California, San Francisco Institutional Review Board (IRB). The IRB determined that this study posed minimal risk and was exempt from a full review.

In sending invitations to key informants as well as at the start of each interview, the researchers explicitly articulated the aims of the study and the data collection methodology. The key informants were free to decline the invitation or withdraw at any point during the interview. All transcribed responses were de-identified to uphold respondents’ confidentiality.

## Results

### Literature review and interviews

1890 titles and abstracts were found through database searches. After removing 829 duplicates, 484 full-text articles were retrieved and reviewed, and 119 were included in this paper. 139 additional documents, reports and websites cited throughout this paper were identified through online searches or provided by key informants. It was found through the literature reviews that only very few articles were on malaria elimination specifically, thus this paper drew from the malaria control literature or from the experiences of other disease programmes where relevant.

Of the 36 domain experts invited as key informants, 29 responded and were interviewed. Interviews in person, over the phone or through video teleconferencing were 46 min on average (range 33–60). Three interviews were done over several e-mail exchanges when the interviewers and key informants could not agree on a common time and date and method of communication. Key informants included researchers from North America, Europe, Africa and Asia (7 out of 29) whose work focused on private sector healthcare delivery systems and financing, programme managers of nongovernmental organizations (NGOs) and implementers working in malaria-endemic countries (17 out of 29), and representatives from the World Health Organization (WHO; 5 out of 29).

### Overview of the private health sector

**Fig. 1:**
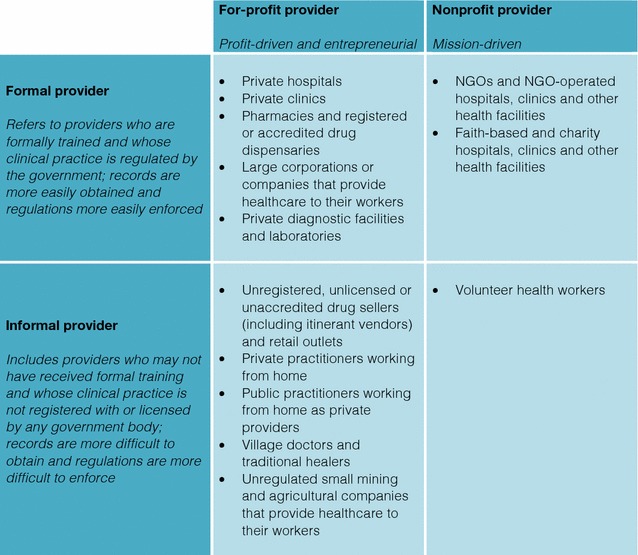
Matrix of private sector healthcare providers

The private health sector includes any outlet, facility or person that provides clinical or diagnostic services and is not managed by a national or local government [3, 17, 45]. As almost all key informants emphasized, the composition of the private health sector varies greatly across countries, but private providers can generally be organized into four groups based on their commercial orientation or business model (for-profit vs nonprofit) and regulation status and/or level of training (formal vs informal) [46, 47]. Based on the literature review and key informant interviews, a private sector matrix was developed (Fig. 1).

Formal private providers have some formal training, accreditation or licensure [48]. The formal for-profit private sector consists of private hospitals and clinics, pharmacies, registered or accredited drug dispensaries and large corporations and companies that provide their own medical services and private diagnostic facilities. Formal nonprofit providers include health facilities owned and operated by NGOs and faith-based groups.

The informal private sector consists of a vast array of outlets run by individuals with little or no formal training [48]. The informal for-profit sector includes unregistered drug vendors and retailers, private and public practitioners who work from home, village doctors and traditional healers, smaller unregulated companies that provide health services, untrained providers and itinerant drug vendors. Volunteer workers fall in the final category of the informal nonprofit sector. Informal providers are responsible for widely varying levels of healthcare interactions in different settings, from 9% in Kenya to 77% in Bangladesh, and are more likely to serve poorer populations and rural areas [49, 50].

Formal providers are often easier to include in national malaria surveillance systems because they are regulated by the government and are typically required to submit records of their services. As all key informants stressed, the informal sector may be more difficult to include because of a lack of regulation or enforcement, making it hard to obtain records in a timely and coordinated manner. Potential legal consequences for operating outside the law may also make such providers hesitant to participate.

The size, contribution and makeup of the private sector in the 35 malaria-eliminating countries varies widely (Additional file 1: Table S1) [51]. In some regions, such as Southeast Asia and some parts of East and West Africa, the private sector is a major, if not the primary, source of healthcare for people across socioeconomic strata. In other regions, such as Southern Africa and some countries in Latin America, the public sector dominates the health system and provides most preventive and curative care.

The types of private providers that are most visited for malaria diagnostic and curative services differ widely by country. In Uganda, private health facilities such as clinics and hospitals are a more popular source of fever treatment than pharmacies, drug stores or general retailers [52]. In contrast, Nigerians are more likely to approach proprietary patent medicine vendors (PPMVs)—a specific type of drug retail outlet in Nigeria that is popular among the poor—for fever treatment compared to any other type of private health facility [53–56]. Similarly, in Benin, Madagascar and Zambia, more people seek fever treatment from general retailers, pharmacies and drug shops than at private health facilities [57–59], whereas in Cambodia, pharmacies and mobile providers are the most common sources of private sector diagnosis and artemisinin-based combination therapy (ACT) [60].

### Key challenges to private sector malaria surveillance

There are many challenges that national malaria programmes must address to ensure proper and timely diagnosis, management and reporting of malaria cases in the private sector. A number of these challenges exist in the public sector as well [23, 61–66], but the mechanisms for resolving them may differ given the accountability public providers have to the government. The following primary challenges with private sector malaria surveillance were identified, focusing specifically on case management and reporting, based on the review of the literature and discussions with key informants.

#### Diagnosis and case management

- Many malaria programmes lack understanding of the composition and market share of the private health sector in malaria testing and treatment. While individual research studies [67–69] and projects like ACTwatch have shed light on the types and quantities of private health providers involved in malaria testing and treatment in many settings, almost half of the key informants stressed that many malaria programmes do not fully know the makeup of the private health sector or public–private split in malaria care in their respective countries. Without this information, ministries of health (MOHs) and malaria programmes cannot craft tailored interventions or policies that match the diversity, size and reach of the private health sector in a particular setting.
- The quality of diagnostic and treatment services varies greatly among private providers and may be quite poor in some settings. Though many patients perceive the quality of care provided in the private sector to be better than in the public sector [70], evidence to support this is lacking [47, 71–76]. Regulatory frameworks that ensure quality of healthcare services may exist but be poorly enforced [77], which may result in poor availability and use of diagnostics [78, 79], over-prescription or overpricing of drugs [80–82] or inappropriate and substandard treatment through ineffective first-line drugs, counterfeit artemisinin-based combinations, artemisinin monotherapies (AMTs) and poor case management [83–91], which are challenges raised by a quarter of the key informants. Limited evidence suggests that these practices might be driven in part by the insistence of community members to receive anti-malarials they are familiar with and are used to taking [92, 93].
- Private providers are often excluded from the design, planning and implementation of public sector disease programmes [94]. Roughly three quarters of the key informants highlighted the fact that national malaria programmes are established by the public sector with public providers in mind, usually with little consideration of how to include the private sector [61]. Despite myriad opportunities for public–private partnerships (PPPs) in malaria testing and treatment [95, 96], PPPs are rare, particularly in malaria elimination settings [97].
- New regulations and protocols may not be communicated to all private providers or, if they are, providers may ignore them [98–101]. As half of the key informants explained, changing national government regulations pose a challenge for many private providers who need to keep up with treatment guidelines [85, 102–104]. Additionally, many informal providers and some formal providers work to some degree outside the law [18], such as selling medicines they are not allowed to sell and without prescriptions.
- Private providers may operate in remote areas far from formal health centers. The distance between public providers and the public sector, four of the key informants argued, may inhibit coordination and communication between the two. At the same time, the private sector including extractive and agricultural industries may be better positioned logistically and financially to provide care in areas where high-risk, vulnerable or hard-to-reach populations reside [32, 97, 105].
- The goals of the national malaria programmes and private providers may differ. According to two-thirds of the key informants, private providers may not be motivated to correctly diagnose or report all malaria cases because of a potential loss in revenue from anti-malarial drug sales [64, 106, 107]. In fact, a profit-driven model may encourage private providers to overprescribe anti-malarials or sell ineffective anti-malarials that patients demand from them [92, 93]. In malaria elimination settings, private providers may be even less likely to purchase and stock malaria diagnostic supplies like rapid diagnostic tests (RDTs) given the dwindling number of cases. National malaria programmes in contrast are primarily interested in ensuring accurate case management and reporting.
- The informal private sector is particularly difficult to address due to its size and lack of organization and government engagement [108]. In some settings there may be an association of private sector providers that represents a large group, and in other settings private providers may be linked through social franchises. However, over three-quarters of the key informants argue that there may be hundreds of private sector providers, including pharmacies and drug vendors, who provide huge volumes of treatment but are not linked with one another and whose practices are often poorly understood [77, 109]. Thus, finding an effective incentive for engagement or authority for enforcement can be a challenge.

#### Case reporting

- Unregulated private providers often do not report into national health management information system (HMIS). Private providers are often excluded from routine disease reporting systems such as HMIS [110], which hampers efforts to rapidly identify and respond to malaria cases, especially in elimination contexts. This was a challenge raised by almost all the key informants.
- Many private providers do not recognize or value the need to count and report all cases [111]. Drug retailers, for example, may not think in terms of numbers of cases but instead may be concerned primarily with product sales. Key informants who are experts in disease surveillance also point out that when case data are collected and provided to the public surveillance system, ensuring the data are meaningful and accurate can be difficult.
- A large proportion of private providers have limited training in accurate diagnosis, prescribing and reporting. Training programmes can be designed to address this [64, 104], but turnover of private providers create a challenge to training programmes. In addition, literacy and numeracy issues among some private providers may hamper training efforts.

### Engagement strategies for malaria diagnosis and case management in the private sector

Developing strategies to engage the private health sector, either through incentive schemes, greater communication, training or policy, is critical for ensuring high quality diagnosis and case management for malaria. However, only limited operational and implementation research has been conducted to guide how to effectively engage the private sector, especially informal providers, across a variety of conditions [97, 112]. Several experts have documented the primary available strategies for incorporating private sector providers to achieve positive child health outcomes across a range of conditions, which include (1) contracting private providers for specific services, (2) social marketing, (3) regulation and standard setting and (4) information dissemination or training [5, 10]. Here, these strategies are discussed, as well as social franchising, as they apply to malaria diagnosis and case management.

Contracting private providers includes utilizing public funds to engage private entities to deliver specific types, qualities and quantities of services which may improve the transparency and efficiency of publicly funded services [113, 114]. However, there is limited evidence on the effectiveness of contracting for malaria services. Contracting of health services to NGOs in Senegal and Madagascar increased coverage of nutritional services and decreased malnutrition rates, notably in areas where public care was less available [97, 115, 116]. In Cambodia, contracting replaced existing public services and resulted in improved access by the poor and lower rates of childhood diarrhea [117]. In Suriname, an NGO called the Medical Mission was funded directly by the Global Fund to Fight AIDS, Tuberculosis and Malaria to provide case management, among other malaria interventions, targeted specifically to high-risk areas with limited access to health services, such as remote gold mining areas. This included training local malaria service providers in delivering free diagnosis and treatment. This programme coincided with a dramatic decline in incidence [118].

Difficulties with contracting include its limited ability to scale, its lower cost-effectiveness compared with government services, increased inequity in service delivery at times, and government’s limited capacity to manage contracts and financial sustainability [114, 119, 120]. However, a review of ten health contracting experiences found that these challenges can be mitigated [119, 121]. A subsequent review found that contracting can improve health system access, though its potential for improving equity, quality and efficiency of health service delivery depended on how well-targeted the programmes were and if quality was adequately monitored and directly linked to payment [122]. Contract management by government agencies is a potential challenge and can be costly, but must be balanced against potential improvements in service delivery. In the case of Cambodia, contracted childhood health services ultimately achieved better outcomes with similar amounts of financial inputs [122]. Financial sustainability is a remaining concern subject to government and donor financing, but in these examples initial contracts were either continued or expanded.

Social marketing or commercialization of health products is used to expand delivery of key interventions to target populations and requires that interventions or services become more profitable and therefore appealing to private providers [5, 97]. This frequently involves a substantial subsidy on the marketed product so that they are affordable for consumers and also profitable for private providers and are therefore more likely to be sold [123]. A prominent example for malaria case management is the subsidization of ACT medicines in the private and public sectors through the Affordable Medicines Facility-malaria (AMFm) programme [124–127] which has shown varying success in improving access to and affordability of frontline anti-malarials and crowding out of AMTs [128–138]. Similar programmes, including randomized trials in Kenya and Tanzania and a pilot study in Uganda whose results informed the AMFm, have also shown that distribution of subsidized ACT medicines paired with training and community education can improve stocking and availability of ACT and the likelihood of being treated with ACT [23, 123, 138–146]. Detailed understanding of the complex distribution chains for anti-malarials is crucial to achieving success of subsidized ACT, as is understanding the role of consumer demand in determining supply [89]. Social marketing strategies have also been used extensively to increase demand and use of insecticide-treated bed nets (ITNs) and to promote hand washing practices [5]. However, the effectiveness of these strategies varies [123]. Social marketing was not able to achieve equity consistently and reach those most at-risk populations in selected projects involving ITNs [147, 148] and anti-malarials through the AMFm in some study sites [149, 150].

Evidence around the ability of social marketing to generate demand for and improve access to RDTs is growing but remains limited to a number of settings [48, 92, 151–158], leaving overtreatment a persistent challenge for many countries that may have successfully expanded access to anti-malarials [16, 79, 127, 128]. Combining training with financial incentives may be an optimal approach to improving diagnosis and appropriate treatment. A recent evaluation of incentives schemes for the use of RDTs by informal private providers in Myanmar compared combinations of price subsidies, financial incentives, and intensified information, education and communication (IEC), and found that a price subsidy combined with intensified IEC resulted in the highest uptake in use and quality of care [154]. Indeed, several reviews suggest subsidization schemes for RDTs would be wise to learn from the experience of ACT and ensure complementary activities such as community awareness, behaviour change communication (BCC) campaigns and training and supervision of providers are integrated with financial mechanisms [137, 157].

Social franchising is a related mechanism for linking private providers to provide and market socially desirable goods [159]. Social franchising has been successfully employed in a number of settings whereby networks of private sector providers are connected through formal agreements, the end result of which is social in addition to financial gain [97, 159, 160]. In this mechanism individual providers are incentivized to join a network of franchises through the creation of brand identity, mass marketing campaigns, access to commodities below market rates and trainings. Throughout their engagement, franchised providers are trained and supported to provide specific services and given access to data and feedback to improve their practices [160]. In Myanmar, providers who joined the Sun Quality Health (SQH) network cited social responsibility for serving the poor as a primary motivation [161, 162]. Social franchising through this network has resulted in more clients because of the perception that these SQH providers provide higher quality care and effective and affordable drugs [161]. However, a systematic review found that while social franchising globally has been shown to increase client volume and client satisfaction, it has had mixed results in terms of healthcare utilization and impact [163].

Regulation through the establishment of laws, policies and standards is a potential means to ensure appropriate case management and reporting in the private health sector, but requires ongoing monitoring to verify that standards are followed [97, 164]. Regulation includes creating specific diagnosis and treatment protocols, creating price controls for health services, regulating pharmaceuticals and essential drug lists, regulating private insurance, executing drug quality standards and involving private providers in establishing standards [10, 63, 164–166]. Licensing and accreditation of private providers is another form of private sector regulation that has shown to successfully improve the supply of ACT medicines among drug sellers and drug shops in Tanzania [166–170]. However, developing countries often allocate insufficient resources to enforce regulations. Often there are gaps in regulatory frameworks, such as infrequent inspections, lack of effective sanctions and lack of enforcement on the part of local regulatory staff, despite awareness of regulation infractions [77, 171, 172]. As Additional file 2: Table S2 shows, regulation over malaria surveillance in elimination settings is largely present, with a few exceptions.

Training of private sector providers may be one of the more operationally feasible and cost-effective approaches to improving private sector case management and has been shown to improve adherence to national guidelines for anti-malarial prescription and improved prescription behaviors by private practitioners in integrated management of childhood illnesses [5, 15, 173–175]. In limited examples, training among drug sellers in Kenya and Tanzania has been associated with improved stocking and selling of recommended anti-malarials and giving the right advice to patients [144, 176, 177]. In Myanmar, IEC was found to be the most cost-effective approach for improving uptake of RDTs by informal providers [178]. Training and provision of subsidized commodities, such as ACT medicines and RDTs, is also commonly reported by providers as a means to improve the stature of their business and empower them to provide better care [179, 180]. Private health providers can also be included in the design and implementation of training, encouraging engagement and ensuring that the trainings address the needs of the intended audience. However, reviews of training interventions reveal that training alone may be insufficient if market-based strategies aligned with a provider’s incentives are not employed at the same time [181]. Combinations of interventions that reinforce each other are likely the most effective approach [182], and effectiveness is likely greatest when training is ongoing and integrated with social marketing approaches, referral systems and increased local regulatory oversight [181].

### Engagement strategies for case reporting in the private sector

Several mechanisms have been employed in different settings to integrate private providers to disease reporting systems. In most countries, formal healthcare providers and facilities such as clinics, hospitals and laboratories that are registered, accredited or monitored by a country’s MOH are required to submit routine statistical reports on specified indicators to a local or national authority [183].

In some countries, certain private for-profit and nonprofit providers are seamlessly integrated into the national HMIS. For example, in Zambia health facilities run by the Churches Health Association of Zambia (CHAZ)—a group of Christian organizations that provides over 35% of healthcare in the country and implements malaria control activities in 22 districts—and other large hospitals submit monthly morbidity and mortality data to the national HMIS [184, 185]. Angola, Kenya, South Africa, Tanzania, Uganda, Zimbabwe and other countries also receive some HMIS data from the private sector, but reporting completeness among private health facilities is variable [186–191]. Limited evidence from elimination settings suggests that reporting may be low. In South Africa, studies have shown that only 26% of malaria cases diagnosed in the private sector are being reported [186, 192]. In Swaziland, while the majority of private providers reported to the HMIS, only half reported to the immediate disease notification system (IDNS) [193].

Disease-specific surveillance systems are a model for integrating the private sector into reporting [187, 188, 190, 194, 195]. Many of these surveillance systems were established through donor funding and are now run by MOHs. For example in Kenya, the National AIDS/STD Control Programme has successfully engaged the private sector in reporting by tying the release of key commodities to case reporting [188]. At the same time the Division of Malaria Control at the Kenyan MOH runs a separate surveillance system with five facilities in epidemic-prone areas reporting regularly on disease burden [188]. However, in some cases these parallel surveillance systems cause fragmentation due to different reporting formats and timeframes and strain limited human resources available to manage these reporting systems [190].

While standalone tuberculosis (TB) surveillance systems still exist, most countries have included TB surveillance in their national HMISs where the private sector is an active participant [196]. This was primarily driven by the adoption of the Stop TB Strategy in 2006 which clearly specified that countries must engage all providers that provide TB care, including those in the private sector [197]. Several public–private mixes (PPMs) were established between national TB programmes and private providers that not only addressed appropriate treatment of TB but also integrated case reporting. As part of a PPM project in Cambodia, a short message service (SMS) malaria reporting and referral system has been implemented among private sector healthcare providers in Pailin Province, with early results suggesting a high proportion of referrals reached the public sector.

Another common method of capturing private sector surveillance is to establish a list of notifiable diseases. A notifiable or reportable disease is any disease or medical condition that medical providers and laboratories are required by law to report to local or national authorities [198]. Most notifiable diseases are rare or vaccine-preventable communicable diseases, such as polio, measles and malaria in elimination settings, but a number of non-communicable diseases such as cancer are reportable to cancer registries. The list of notifiable diseases varies from country to country, and in countries with decentralized health systems, different states or provinces may establish their own list of notifiable diseases while still adhering to national guidelines [199–201].

Making a disease notifiable can improve surveillance, but it is not a perfect system. Many countries have documented varying levels of reporting completeness (with completeness rates ranging from 2 to 95% in different studies) and timeliness among public and private providers, both of which are critical for disease control and elimination [198, 202–208]. Several barriers have been identified in reporting notifiable diseases, including poor physician knowledge about which diseases are notifiable, poor understanding of the proper procedures when reporting a disease and the significant amount of time required to fill out the forms for disease reporting [110, 111, 209–219].

To address these barriers, a number of interventions have been implemented, including posting a condensed list of notifiable diseases in health facilities, making reporting forms widely available, conducting disease reporting trainings and providing regular feedback to providers about how their data is used [201, 213, 214]. The use of electronic or Internet-based reporting systems has been shown to improve completeness and timeliness of reporting in many settings [201, 220]. Appropriate incentives can promote consistent and timely reporting, such as in Taiwan where notifiable disease reporting is linked to national health insurance reimbursements and a small remuneration and has significantly improved reporting completeness and timeliness [221, 222]. In Swaziland, providers reported willingness to report into the malaria surveillance system without additional incentives, but requested improved training on how to use reporting forms and frequent follow up and interaction with malaria programme officers [193]. However, research on improving reporting for informal private providers remains an important gap across all settings.

#### Country case studies

Table 2 summarizes the approaches used by six focus countries in engaging the private sector in malaria surveillance and the successes and challenges faced by their malaria programmes in employing these strategies. The countries studied in-depth include two malaria-eliminating (Swaziland and Vietnam) and four neighboring countries (Cambodia, Mozambique, Myanmar and Zambia) in Southeast Asia and Southern Africa that either have sub-national elimination goals or are part of regional networks with elimination goals. Additional file 3 includes a longer narrative on each of these six country case studies.

**Table 2:** Summary of findings from country case studies

Country	Malaria burden (2013) [223]	National or regional elimination goal	Private sector utilization	Private sector engagement strategies for malaria			
				Diagnosis and case management	Successes and challenges	Case reporting	Successes and challenges
Vietnam	35,406 cases and 6 deaths	National elimination by 2015	No malaria-specific data available; over 60% of all outpatient care provided by private sector (as high as 80% for TB care) [224–226]	Regulation by government	Though Vietnam has an active social franchise network, it currently does not work with the NMCP Engagement strategies have been employed in other programme areas and may serve as guide for malaria (e.g., total market approach for contraceptives, social franchising of private clinics, and TB PPPs)	None	Malaria Information System does not include private providers Experience with TB PPPs and private sector reporting of cases can be used as model
Cambodia	24,130 cases and 12 deaths	Asia Pacific regional elimination by 2030	70% of malaria patients seek care in private sector; 75% of malaria treatment received from private sector [227]	Regulation by government Provider training Social marketing Social franchising	Increased crackdown on illegal drug outlets and establishment of drug inspection police to identify private pharmacies selling AMTs Successful rollout of prepackaged, quality-assured ACT (i.e., Malarine) Use of outlet survey results to guide policy formulation and interventions Strengthened referral linkage between public and private providers through SMS system in pilot areas only Trainings for private providers and regular meetings between public and private sectors in selected provinces Private sector remains largely unregulated, particularly drug sellers and village vendors Incentives needed to ensure proper testing and treatment even as cases decline	HMIS integration	SMS system tracks private sector referrals to public facilities (in pilot areas only)
Myanmar	333,871 cases and 236 deaths	Asia Pacific regional elimination by 2030	36% of malaria patients seek care in private sector; 65% of malaria treatment received from private sector [228, 229]	Regulation by government Provider training Social marketing Social franchising	Services and commodities sold by providers from two social franchise networks are regularly monitored and improved Distribution of RDTs and provider training as part of the Myanmar Artemisinin Resistance Containment project Successful rollout of Artemisinin Monotherapy Replacement project to increase quality-assured ACTs in private sector	None	NGOs and private providers not formally integrated with HMIS, although changes are underway
Swaziland	669 cases and 4 deaths	National elimination by 2015	No malaria-specific data available but private care minimal according to key informants	None	No law for government oversight of private sector exists NMCP and partners have explored and addressed barriers to proper case management of malaria in the private sector; government and private providers have established communication channels	Notifiable or reportable disease list HMIS integration Provider trainings	Reporting malaria to HMIS and IDNS mandatory for all providers In an effort to improve reporting rates, NMCP staff visit private providers and provider training on IDNS
Mozambique	3,924,832 cases and 2941 deaths	Southern Africa regional elimination by 2030	No malaria-specific data available but private care minimal according to key informants; malaria testing services available in private sector but not ACT	Regulation by government	Unofficial partnerships between government and private companies (particularly extractive industry) exist	Notifiable or reportable disease list	No existing channels for routine reporting of malaria data among private providers No law for mandatory reporting in place, therefore enforcement is poor
Zambia	5,465,122 cases and 3548 deaths	Southern Africa regional elimination by 2030	7–10% of malaria patients seek care in private sector; 12–20% of malaria treatment received from private sector; [59] proportions are larger when church-run facilities, which provide 35% of all healthcare services, are considered [230, 231]	Regulation by government Accreditation of providers	Registered and licensed private drug shops allowed to stock and sell ACTs, based on positive findings of Zambia Access to ACTs Initiative	HMIS integration	Many small private clinics, health facilities, pharmacies, and shops excluded from national and district HMIS

*ACT* artemisinin-based combination therapy, *AMT* artemisinin monotherapy, *HMIS* health management information system, *IDNS* Infection Diseases Notification System, *NGO* nongovernmental organization, *NMCP* National Malaria Control Programme, *PPP* public–private partnership, *RDT* rapid diagnostic test, *SMS* short message system, *TB* tuberculosis

The most common private sector engagement strategy employed across the six focus counties to improve malaria diagnosis and case management is some form of regulation by governments. The only exception is Swaziland where legislation that gives the public sector authority to oversee private providers and their activities is still lacking. Despite this, the Swazi National Malaria Control Programme (NMCP) in collaboration with the Clinton Health Access Initiative has explored and addressed barriers to proper case management and reporting of malaria cases in the private sector.

Social marketing has also been used successfully in Cambodia and Myanmar to rollout RDTs and quality-assured ACT medicines in the private sector. Coupled with provider and patient education activities and a ban on AMT importation, giving private providers access to subsidized ACT medicines crowded out less effective AMTs in the market that were exacerbating artemisinin resistance. As described earlier, social franchising has been implemented successfully in Myanmar, leading to improved perception of their services and high rates of testing and treating for malaria.

In terms of case reporting, most countries have tried to integrate private providers to HMIS; however, participation has been variable. In Zambia, large private hospitals routinely submit case data to the HMIS while many small private clinics are left out. In Vietnam and Myanmar, there are no formal mechanisms for collecting or receiving malaria case data from private providers. The PPM pilot in Cambodia suggests that if integrated with training, regulation, and recognition, SMS reporting may be a simple way to include private providers in routine reporting and referral of malaria cases.

## Discussion

This review combined expert opinions and published and grey literature. Until recently, research and data on private sector surveillance and case management for malaria elimination settings have been limited, especially for the informal sector, and for reporting schemes in particular. While numerous countries have piloted strategies to engage the private sector, systematic evaluations of these activities are rare, which makes it difficult to provide general evidence-based recommendations. As a result, the available and recommended strategies are highly dependent on country context and a mix of activities is likely required in most settings. Opportunities for and means of including the private sector in national malaria elimination strategies will necessarily vary by setting. Despite these nuances, the review of the literature on private sector engagement strategies in all malaria endemic countries, and discussions with experts, suggests that several general lessons that can be applied.

First, if they have not already, country programmes and partners should conduct research to understand how to effectively engage the private sector in each setting. All health systems are mixed, and private sector provision of healthcare is a reality everywhere and methods of engagement need to be better understood. All malaria eliminating countries should, at a minimum, conduct a landscaping effort to understand the breadth and quality of private sector diagnosis, treatment and reporting and identify gaps and challenges in their countries. Landscaping exercises can be localized to malaria endemic and/or highly vulnerable and receptive areas. This effort should lead to a prioritization of specific provider groups to work with, such as private sector providers that regularly interface with high-risk groups for malaria or other populations that are typically missed by the public sector. The landscaping effort should lead to a collaborative multi-stakeholder discussion of appropriate engagement strategies.

Second, where feasible, programmes should aim to facilitate linkages and routine interaction between the national malaria programme, public providers and private providers. Linkages can be established through PPM systems, referral systems, direct contracting or regular shared trainings. At a minimum, regular meetings at provincial or district levels will help to build relationships and trust and will enable private providers to see themselves as valuable partners who are an essential piece of the elimination process. Ideally, human and financial resources should be dedicated to support these relationships. It will not be possible to include all private providers in these interactions, but the landscaping of the private sector that will help identify the key private provider groups in each setting. One initial manner to engage for-profit providers in malaria surveillance may be to track diagnosis and treatment flows through a limited sample of sentinel providers rather than on a national scale.

Third, based upon discussions with key provider groups, determine appropriate and effective incentives and disincentives to encourage appropriate diagnosis, treatment and reporting of malaria by private providers. Incentives, while not necessarily financial, will have to address a financial need if they are to be sustainable. Social franchising and social marketing are potentially effective incentive schemes to align the goals of population health with the interests of private providers. Pay-for-performance schemes may allow for improved quality and cost-effectiveness for directly contracted services. Punitive incentives, or disincentives, such as refusing renewal of registration or licensure or restricting access to subsidized commodities or government support to providers who have not reported consistently may be an alternative or additional approach.

Fourth, country programmes with a large informal private sector should consider investing in schemes to provide opportunities for accreditation of informal private providers. Providers that are able to reach agreed standards should be allowed to operate legally and should be regulated to those standards, including the provision of surveillance information, and those that are unable to attain these standards need to be shut down. Standards for informal providers need to be realistic enough to be achieved and the vested interests of larger pharmacies carefully considered. Formalizing these institutional relationships is a key step to fully integrate public and private healthcare delivery under a regulatory framework that can be managed by governments. In some cases, using a strong intermediary presence such as a large NGO or professional body or association can help manage the public and private relationships.

Fifth, malaria programmes should attempt to ensure frequent trainings of private providers, or inclusion of private providers in routine public sector training events. Trainings can provide private providers with updates to regulations and guidelines and will allow an opportunity to discuss the challenges they face; when combined with other interventions such as social marketing schemes, routine training has the potential to improve the cost-effectiveness and sustainability of these interventions. Trainings should build up to accreditation and then be managed through quality assurance processes to ensure standards are met and maintained.

Sixth, countries approaching malaria elimination should make malaria a notifiable disease. Making malaria a notifiable disease will ensure, at a minimum, a framework for mandatory reporting by all providers who diagnose and treat malaria. Additionally, as countries approach malaria elimination, programmes should increase restrictions on providers that are legally permitted to provide malaria diagnosis and treatment.

Seventh, provide simple and inexpensive reporting and referral systems for the private sector. Reporting and referral systems may include SMS reporting, web-based platforms or other convenient and easy-to-use systems. Training on how to use the reporting referral systems and how to ensure quality data collection must be included in this process. It may not be possible to include all private providers in the reporting and referral system, therefore knowing which private providers to engage in each country and district, and potential incentive structures, will help prioritize this process.

Finally, country programmes and partners should share lessons of successful strategies across borders through regional assessments and engagement. Private sector challenges and opportunities for engagement in malaria surveillance are often similar across countries in a given region. Regional bodies and meetings allow a venue for sharing lessons across borders and engaging large multi-national actors.

## Limitations

This review focused primarily on existing challenges and potential solutions for improving case management and reporting for malaria in the private sector. While documenting the distribution chain is an important component of ensuring appropriate and affordable commodities for case management, for brevity commodity distribution chain analyses were not emphasized in the literature review and discussion with key informants.

## Conclusion

While numerous opportunities exist to address private sector engagement in malaria diagnosis, treatment and reporting, strategies need to be tailored to each country’s unique political, economic and epidemiologic context. Further research is needed to determine the most effective methods for improving uptake of diagnostics and rapid reporting and referral of malaria cases by informal private providers. Knowledge sharing between countries and collaborations that include private sector healthcare providers are essential to building consensus on effective approaches. As demonstrated in this review paper, although there is substantial awareness that private providers in many settings are already doing much of this work effectively, improved efforts to include them in formal national processes are crucial to achieving and maintaining malaria elimination.

## Additional files

**Additional file 1.** **Table S1.** Private sector size and utilization in 34 malaria-eliminating countries.

**Additional file 2.** **Table S2.** Regulation of private sector malaria surveillance in 34 malaria-eliminating countries.

**Additional file 3.** Case study narratives.

## Abbreviations

ACT: artemisinin-based combination therapy
AMFm: Affordable Medicines Facility-malaria
AMT: artemisinin monotherapy
BCC: behaviour change communication
CHAZ: Churches Health Association of Zambia
HMIS: health management information system
ITN: insecticide-treated bed net
IEC: information, education and communication
IDNS: Infection Diseases Notification System
MOH: Ministry of Health
NGO: nongovernmental organization
NMCP: National Malaria Control Programme
PPM: public–private mix
PPP: public–private partnership
PPMV: proprietary patent medicine vendor
RDT: rapid diagnostic test
SMS: short messaging system
SQH: Sun Quality Health
TB: tuberculosis
WHO: World Health Organization
