# Supplementary material for: Engaging the private sector in malaria surveillance: a review of strategies and recommendations for elimination settings
Source: Malar J. 2017 Jun 14;16:252. doi: 10.1186/s12936-017-1901-1 (PMC5471855; doi:10.1186/s12936-017-1901-1)
Supplement: Supplementary file 1 — Additional file 1. Table S1. Private sector size and utilization in 34 malaria-eliminating countries. [file 12936_2017_1901_MOESM1_ESM.docx]

**Table S1**. Private sector size and utilization in 34 malaria-eliminating countries^*^

| **Country** | **Relative Size of the Private and Public Sectors**^†^ | | **Private Sector Utilization** | | | |
| --- | --- | --- | --- | --- | --- | --- |
|  | *Number or proportion of formal private providers or health facilities* | *Number or proportion of formal public providers or health facilities* | *Among children under 5 years who sought treatment for fever, the proportion of individuals who received care from a private provider or health facility* | *Among children under 5 years who sought treatment for fever, the proportion of individuals in the lowest wealth quintile who received care from a private provider or health facility* | *Among children under 5 years who sought treatment for fever, the proportion of individuals in the highest wealth quintile who received care from a private provider or health facility* | *Proportion or number of malaria cases diagnosed by private providers or in private facilities*^‡^ |
| **Algeria** | 20% of physicians^1^ | No data | No data | No data | No data | No data |
| **Azerbaijan** | No data | 1450 healthcare facilities (2010)^2^ | 4.36 (95% CI 1.74 - 10.50)^3^ | No observations^3^ | No observations^3^ | No data |
| **Belize** | 4 hospitals and 69 outpatient centers and 25% of health professionals (2007)^4^ | 7 hospitals and 44 outpatient centers and 75% of health professionals (2007)^4^ | No data | No data | No data | No data |
| **Bhutan** | Almost zero^5^ | Majority^5^ | No data | No data | No data | No data |
| **Botswana** | 650 general practitioners (2013)^6^ and 6 private hospitals and 167 medical clinics (2008)^7^ | 683 general practitioners (2013)^6^ and 34 hospitals and 1,499 clinics and health posts (2008)^7^ | No data | No data | No data | No data |
| **Cape Verde** | 60 medical practices, 31 pharmacies, and 15 laboratories^8^ | 2 national reference hospitals, 3 regional hospitals 30 health centers, 34 health posts, 113 basic health units^8^ | No data | No data | No data | No data |
| **China** | 8440 hospitals (2011)^9^ and 133,132 clinics (2005)^10^ | 14,328 hospitals (2011)^11^ and 201,509 clinics, infirmaries, and health centers (2005)^10^ | No data | No data | No data | 80%^12^ |
| **Costa Rica** | 38.4% of health professionals (2008)^13^ | 61.6% of health professionals (2008)^13^ | No data | No data | No data | 50%^14^ |
| **Democratic People's Republic of Korea** | No data | 300,000 health workers^15^ | No data | No data | No data | No data |
| **Dominican Republic** | 8.30% (2007)^16^ | 91.70% (2007)^16^ | 23.65% (95% CI 19.74 - 28.07)^17^ | 5.76 (95% CI 3.86 - 8.5)^17^ | 31.36% (95% CI 20.56 - 44.64)^17^ | No data |
| **El Salvador** | Exact number not known (2010)^18^ | 619 health facilities (2010)^18^ | No data | No data | No data | No data |
| **Iran** | No data | No data | No data | No data | No data | No data |
| **Guatemala** | 6,963 health facilities or 81% of total (2011)^19,20^ | 1,617 health facilities (2011)^19,20^ | No data | No data | No data | No data |
| **Honduras** | 60 hospitals (2009)^21^ | 30 hospitals (2009)^21^ | 17.88% (95% CI 16.11 - 19.80)^22^ | 5.11% (95% CI 3.76 – 6.89)^22^ | 60.34 (95% CI 53.04 - 67.21)^22^ | No data |
| **Malaysia** | 10,382 physicians or 34% (2009)^23^ | 20,154 physicians or 66% (2009)^23^ | No data | No data | No data | No data |
| **Mayotte** | No data | No data | No data | No data | No data | No data |
| **México** | 42.6% health employees (2012) | 57.4% health employees (2012) | No data | No data | No data | No data |
| **Namibia** | 0.20 per 10,000 population or 53% of all healthcare workers (2007; 72% of doctors, 46% of RNs, 89% of pharmacists, 53% of pharmacist assistants, and 70% of social workers)^24^ | 0.88 per 10,000 population or 47% of all healthcare workers (2007; 28% of doctors, 54% of RNs, 11% of pharmacists, 47% of pharmacist assistants, and 30% of social workers)^24^ | No data | No data | No data | No data |
| **Nepal** | 162 hospitals (2008)^25^ or as much as 78% of hospitals^26^ and 6,888 doctors (2011)^27^ | 96 hospitals (2008)^25^ or 12% of hospitals^26^ and 1,447 doctors, 6,552 nurses, 7,559 paramedics, 4,289 public health workers, and 785 alternative medicine practitioners (2011)^27^ | 53.56% (95% CI 40.94 - 52.02)^28^ | 18.42% (95% CI 10.89 - 29.44)^28^ | 76.99% (95% CI 67.58 - 84.31)^28^ | No data |
| **Nicaragua** | N/A | 2,530 health facilities^2^ | 2.89% (95% CI 1.96 - 4.26)^29^ | No observations^29^ | 84.16% (95% CI 67.17 - 93.24)^29^ | No data |
| **Panamá** | No data | No data | No data | No data | No data | No data |
| **Paraguay** | 1126 health facilities (2007)^30^ | 984 health facilities (2007)^30^ | No data | No data | No data | No data |
| **Philippines** | 70% of all health professionals (2011)^31^ | 30% of all health professionals (2011)^31^ | 27.66% (95% CI 24.30 - 31.30)^32^ | 11.47% (95% CI 7.76 - 16.64)^32^ | 27.56% (95% CI 21.43 - 34.67)^32^ | No data |
| **Republic of Korea** | 90% of all medical institutions (2006)^33^ | 10% of all medical institutions (2006)^33^ | No data | No data | No data | No data |
| **São Tomé and Príncipe** | Almost none^34^ | Majority^34^ | 22.98% (95% CI 16.00 - 31.83)^35^ | 16.66% (95% CI 7.82 - 32.02)^35^ | 19.87% (95% CI 9.16 - 37.89)^35^ | No data |
| **Saudi Arabia** | 7.59 physicians, 9.72 nurses, 0.34 pharmacists, and 3.48 allied health professionals per 10,000 population (2012)^36^ | 2.87 physicians, 5.59 nurses, 0.05 pharmacist, and 3.46 allied health professionals per 10,000 population (2012)^36^ | 66% of outpatient visits^36^ | No data | No data | No data |
| **Solomon Islands** | 4 private hospitals (2012) ^37^ | 8 public hospitals, plus 187 nurse aid posts, 102 rural health clinics, and 38 area health centers (2012)^37^ | No data | No data | No data | No data |
| **South Africa** | 6,702 physicians or 60% (2002)^38^; 216 private hospitals (2010)^39^ | 4,468 physicians or 40% (2002)^38^; 393 hospitals (2012)^39^ | No data | No data | No data | No data |
| **Sri Lanka** | 125 hospitals or 17% (2011)^40^ | 592 hospitals or 83% (2011)^40^ | No data | No data | No data | 19%^41^ |
| **Swaziland** | 2 mission hospitals, 53 private clinics, 73 mission facilities, 22 industry owned facilities (2012); 1 medical doctor and 3.6 nurses per 10,000 (2013)^42^ | 6 hospitals, 8 public health units, 5 health centers, 162 clinics, 187 outreach sites (2012); 1.3 doctors and 15 nurses per 10,000 (2012)^42^ | No data | No data | No data | No data |
| **Tajikistan** | 14 hospitals and a number of pharmacies, dental care centers, and small diagnostic facilities (2010)^43^ | 426 hospitals (2007)^43^ | 1.27% (95% CI 0.315 - 5.00)^44^ | No observations^44^ | No observations^44^ | No data |
| **Thailand** | 17% of doctors, 7.2 % of dentists, 14.7% of pharmacists, and 10.7% of nurses (2009)^45^ | 53.5% of doctors, 64.8% of dentists, 73.4% of pharmacists, and 73.4% of nurses (2009)^45^ | No data | No data | No data | 0% (antimalarials banned in private sector)^46^ |
| **Turkey** | 19.5% of physicians, 85.0% of nurses, 60.6% of dentists, and 34.0% of all hospitals (2010)^47^ | 80.5% of physicians, 15.0% of nurses, 39.4% of dentists and 66.0% of hospitals in (2010)^47^ | No data | No data | No data | No data |
| **Vanuatu** | 1 international health center, 6 medical clinics, 4 pharmacists, 1 physiotherapy clinic, 1 dental clinic, 2 laboratory, and 4 counseling centers (2010)^48^ | 231 aid posts, 89 dispensaries, 37 health centers, 8 municipal clinics, 2 referral hospitals, 4 provincial hospitals in (2010)^48^ | No data | No data | No data | No data |
| **Vietnam** | 11.5 per 10,000 population (2005)^49^ | 6.7 per 10,000 population (2005)^49^ | No data | No data | No data | No data |

******95% CI* – 95% confidence interval; *No data* – no data found from published surveys (i.e., Demographic and Health Surveys, Malaria Indicator Surveys or ACTwatch surveys); *No observations* – no reported respondents (n=0) from specific survey referenced.

†The types of private and public providers reported vary by country and by source of data; thus, these numbers should be interpreted in that limited context. Most reports include formal providers only. For private providers, both for-profit and nonprofit providers are included.

‡Private facilities in this column include retail outlets that provide malaria diagnostic services.

**References:**

1 Pasanen-Zentz H. Algeria health sector overview. 2011; published online Oct 27. http://www.slideshare.net/FinproRy/algeria-health-sector-overview-heli-pasanenzentz-finpro (accessed Nov 5, 2014).

2 World Health Organization. Baseline country survey on medical devices 2010. 2011. http://whqlibdoc.who.int/hq/2011/WHO_HSS_EHT_DIM_11.01_eng.pdf (accessed Nov 11, 2014).

3 State Statistical Committee of the Republic of Azerbaijan, Macro International Inc. Azerbaijan demographic and health survey, 2006. 2008; published online May. http://dhsprogram.com/pubs/pdf/FR195/FR195.pdf.

4 Pan American Health Organization, Ministry of Health. Health systems profile Belize. 2009; published online July. http://www.paho.org/blz/index.php?option=com_docman&task=doc_view&gid=64&Itemid=237 (accessed Nov 5, 2014).

5 Yangzom T, Gueye CS, Namgay R, *et al.* Malaria control in Bhutan: case study of a country embarking on elimination. *Malar J* 2012; **11**: 9.

6 Banyan Global, Jhpiego, Marie Stopes International, Monitor Group, O’Hanlon Health Consulting. Botswana private health sector assessment. 2013; published online May 18. http://www.shopsproject.org/sites/default/files/resources/Botswana Private Health Sector Assessment.pdf (accessed Nov 5, 2014).

7 World Health Organization Regional Office for Africa. Botswana: the health system. Afr. Health Obs. 2014. http://www.aho.afro.who.int/profiles_information/index.php/Botswana:The_Health_System#cite_note-five-1 (accessed Nov 5, 2014).

8 Global Health Group Project Team, National Malaria Control Program in Cape Verde. Eliminating malaria in Cape Verde. 2013; published online Aug. http://globalhealthsciences.ucsf.edu/sites/default/files/content/ghg/country-briefings/africa/Cape-Verde2013.pdf.

9 PwC. Investing in China’s private healthcare systesm. 2013; published online April. http://www.pwc.com/en_US/us/10minutes/assets/pm-13-0210_10minutes-china-reformed-healthcare-sector-v17.pdf (accessed Nov 5, 2014).

10 Zhen L, Baozhen W, Yun Z. The current situation and analysis of medical and health service regulation in China: background discussion paper to inform the Regulatory Reform Review of China. 2007; published online Sept. http://www.oecd.org/gov/regulatory-policy/39218123.pdf.

11 National Bureau of Statistics of China. China statistical yearbook 2013. 2013. http://www.stats.gov.cn/tjsj/ndsj/2013/indexeh.htm (accessed Nov 5, 2014).

12 Asia Pacific Malaria Elimination Network, Malaria Elimination Initiative. Strategy & intervention matrix: China. 2013; published online July. http://apmen.org/storage/country-partner/updated-cp-matricies-2013/APMEN_Matrix_China_2013Update.pdf (accessed Nov 5, 2014).

13 Perfil de situación de salud de Costa Rica. 2009; published online Sept. http://www.paho.org/cor/index.php?gid=87&option=com_docman&task=doc_view (accessed Nov 5, 2014).

14 Ministerio de Salud Costa Rica. Dirección vigilancia de la salud. 2014; published online March 11. http://www.paho.org/hq/index.php?option=com_docman&task=doc_view&gid=24863&Itemid=.

15 Barrett J. The North Korean healthcare system: on the fine line between resilience and vulnerability. *Resil Interdiscip Perspect Sci Humanit* 2011; **2**: 52–65.

16 Ministry of Health and Social Assistance, Executive Commission for Health Sector Reform, Health Statistics Department, Regional Office of the Pan American Health Organization/World Health Organization. Health systems profile Dominican Republic: monitoring and analyzing health systems change, 3rd edn. Santo Domingo, Dominican Republic: PAHO/WHO, 2007.

17 Centro de Estudios Sociales y Demográficos, Macro International Inc. República Dominicana encuesta demográfica y de salud 2007. 2008; published online May.

18 Acosta M, del Rocío Sáenz M, Gutiérrez B, Bermúdez JL. Sistema de salud de El Salvador. *Salud Pública México* 2011; **53**: 188–96.

19 Lao Pena C. Improving access to health care services through the Expansion of Coverage Program (PEC): the case of Guatemala. Washington, D.C.: The World Bank, 2013 https://wdronline.worldbank.com/handle/10986/13314 (accessed Dec 7, 2015).

20 Ministerio de Salud Pública y Asistencia Social. Diagnóstico nacional de salud. 2012. http://maternoinfantil.org/archivos/smi_D286.pdf (accessed Dec 7, 2015).

21 Pan American Health Organization. Health systems profile Honduras. 2009; published online Feb. http://new.paho.org/hq/dmdocuments/2010/Health_System_Profile-Guatemala_2007.pdf (accessed Dec 7, 2015).

22 Secretaría de Salud, Instituto Nacional de Estadística, ICF International. Encuesta nacional de demografía y salud 2011 - 2012. 2013. http://dhsprogram.com/pubs/pdf/FR274/FR274.pdf (accessed Dec 7, 2015).

23 Jaafar S, Noh KM, Muttalib KA, Othman NH, Healy J. Malaysia health system review. *Health Syst Transit* 2013; **3**: 1–103.

24 Feeley F, de Beer I, Sulzbach S, others. Namibia private sector assessment. 2010. http://www.shopsproject.org/sites/default/files/resources/Final Namibia PSA 4-19-11.pdf (accessed Nov 5, 2014).

25 RTI International. Overview of public-private mix in health care service delivery in Nepal. 2010; published online June. https://www.rti.org/pubs/42_nepal_overviewpublicprivate.pdf (accessed Dec 7, 2015).

26 Raj Mishra S, Khanal P, Kumar Karki D, Kallestrup P, Enemark U. National health insurance policy in Nepal: challenges for implementation. *Glob Health Action* 2015; **8**. DOI:10.3402/gha.v8.28763.

27 Ministry of Health and Population. Human resources for health strategic plan 2011-2015 draft. 2012; published online Jan. http://www.who.int/workforcealliance/countries/Nepal_HRHStrategicPlan_finaldraft.pdf (accessed Dec 7, 2015).

28 Population Division Ministry of Health and Population, New ERA, ICF International. Demographic and health survey 2011. 2012. http://dhsprogram.com/pubs/pdf/FR257/FR257%5B13April2012%5D.pdf (accessed Dec 7, 2015).

29 Instituto Nacional de Información de Desarrollo. Encuesta Nicaragüense de demografía y salud 2011/12 (ENDESA 2011/2012). 2012. http://ghdx.healthdata.org/record/nicaragua-national-demographic-and-health-survey-2011-2012 (accessed Dec 8, 2015).

30 Pan American Health Organization. Health systems profile Paraguay. 2008; published online Nov. http://www.paho.org/hq/dmdocuments/2010/Health_System_Profile-Paraguay_2008.pdf (accessed Nov 5, 2014).

31 Romualdez AG, dela Rosa JFE, Flavier JDA, *et al.* The Philippines: health system review. *Health Syst Transit* 2011; **1**: 1–129.

32 National Statistics Office, ICF Macro. Philippines national demographic and health survey 2008. 2009. http://dhsprogram.com/pubs/pdf/FR224/FR224.pdf (accessed Nov 5, 2014).

33 Chun CB, Kim SY, Lee JY, Lee SY. Republic of Korea health system review. *Health Syst Transit* 2009; **11**: 1–183.

34 Costa C, Santana P, Almendra R, Freitas P, Zaky A. And when there is no information? The case of São Tomé and Principe. In: ICSMM 09. Barcelona, Spain, 2009. http://rcc.gov.pt/SiteCollectionDocuments/article_20091207_en.pdf (accessed Nov 5, 2014).

35 Instituto Nacional de Estatística, MEASURE DHS. São Tomé e Príncipe inquérito demográfico e sanitário 2008-2009. 2014. http://dhsprogram.com/pubs/pdf/FR233/FR233.pdf (accessed Nov 17, 2014).

36 Saudi Arabia Ministry of Health. Health statistics annual book 2012. 2013. http://www.moh.gov.sa/en/Ministry/Statistics/book/Documents/1433.pdf (accessed Nov 5, 2014).

37 World Health Organization, Ministry of Health Solomon Islands. Health service delivery profile Solomon Islands 2012. 2012. http://www.wpro.who.int/health_services/service_delivery_profile_solomon_islands.pdf (accessed Nov 5, 2014).

38 Breier M, Wildschut A. Doctors in a divided society: the profession and education of medical practitioners in South Africa. Cape Town, South Africa: HSRC Press, 2006.

39 Day C, Gray A. Health and related indicators. In: Padarath A, English R, eds. South Africa health review 2013/14. Durban, South Africa: Health Systems Trust, 2014: 201–346.

40 Govindaraj R, Navaratne K, Cavagnero E, Seshadri SR. Health care in Sri Lanka: What can the private health sector offer? 2014; published online June. http://documents.worldbank.org/curated/en/2014/06/20053127/health-care-sri-lanka-can-private-health-sector-offer.

41 Wickremasinghe R, Newby G. Maintaining zero: an update to the Sri Lanka malaria elimination case study. 2014; published online Oct. http://globalhealthsciences.ucsf.edu/sites/default/files/content/ghg/mei-maintaining-zero-sri-lanka.pdf.

42 Ministry of Health and Social Welfare. Human resources for health strategic plan 2012-2017. 2012; published online Oct. www.gov.sz/images/stories/Health/human resources for health strategic plan.doc (accessed Nov 5, 2014).

43 Khodjamurodov G, Rechel B. Tajikistan: health system review. *Health Syst Transit* 2010; **12**. http://www.hpi.sk/cdata/Documents/HIT/Tajikistan_2010.pdf (accessed Nov 5, 2014).

44 Statistical Agency under the President of the Republic of Tajikistan, Ministry of Health, MEASURE DHS. Tajikistan demographic and health survey 2012. 2013; published online Nov. http://dhsprogram.com/pubs/pdf/FR279/FR279.pdf (accessed Nov 17, 2014).

45 Wibulpolprasert S, Sirilak S, Ekachampaka P, Wattanamano N, editors. Thailand health profile report 2008-2010. Thailand: Bureau of Policy and Strategy, Ministry of Public Health, 2011.

46 Bureau of Vector-Borne Disease. National strategic plan for malaria control and elimination in Thailand 2011-2016. 2011. http://apmen.org/storage/thailand_national_strategic_plan_2011-2016.pdf.

47 Tatar M, Mollahaliloğlu S, Şahin B, Aydin S, Maresso A, Hernández-Quevedo W. Turkey: health system review. *Health Syst Transit* 2011; **13**: 1–186.

48 World Health Organization Western Pacific Regional Office, Ministry of Health Vanuatu. Health service delivery profile Vanuatu 2012. 2012. http://www.wpro.who.int/health_services/service_delivery_profile_vanuatu.pdf (accessed Nov 5, 2014).

49 Tuan T, Dung VTM, Neu I, Dibley MJ. Comparative quality of private and public health services in rural Vietnam. *Health Policy Plan* 2005; **20**: 319–27.
