# Supplementary material for: Engaging the private sector in malaria surveillance: a review of strategies and recommendations for elimination settings
Source: Malar J. 2017 Jun 14;16:252. doi: 10.1186/s12936-017-1901-1 (PMC5471855; doi:10.1186/s12936-017-1901-1)
Supplement: Supplementary file 2 — Additional file 2. Table S2. Regulation of private sector malaria surveillance in 34 malaria-eliminating countries. [file 12936_2017_1901_MOESM2_ESM.docx]

**Table S2**. Regulation of private sector malaria surveillance in 34 malaria-eliminating countries^*^

| **Country** | **Private Sector Regulation** | | |
| --- | --- | --- | --- |
|  | *Government agency regulating private providers* | *Regulations for malaria surveillance in private sector (Yes or No)* | *Government requirements for malaria reporting* |
| **Algeria** | Ministry of Health and Population | Yes | Case reporting mandatory^1^ |
| **Azerbaijan** | Ministry of Healthcare | Yes | Case reporting mandatory (since 1930)^1^ |
| **Belize** | Ministry of Health | Yes | No data |
| **Bhutan** | Medical and Health Council | Yes | Case reporting mandatory (since 2012)^1^ |
| **Botswana** | Botswana Health Professions Council | Yes | No data |
| **Cape Verde** | Ordem dos Medicos de Cabo Verde | Yes | Case reporting mandatory^1^ |
| **China** | Ministry of Public Health | Yes | National web-based case reporting system^1^ |
| **Costa Rica** | Ministry of Health | Yes | Case reporting mandatory^1^ |
| **Democratic People's Republic of Korea** | No data | Yes | Case reporting mandatory (since 1999)^1^ |
| **Dominican Republic** | Dominican Medical Board^2^ | No data | No data |
| **El Salvador** | Ministerio de Salud | No^1^ | N/A |
| **Iran** | Ministry of Health and Medical Education | Yes | Case reporting mandatory (since 1949)^1^ |
| **Guatemala** | Ministerio de Salud Pública y Asistencia Social^3^ | No data | No data |
| **Honduras** | Secretaría de Salud | No data | No data |
| **Malaysia** | Ministry of Health | Yes | Case reporting mandatory (since 1975)^1^ |
| **Mayotte** | Agence de Santé Océan Indien | Yes^4^ | Case reporting mandatory^1^ |
| **México** | Secretaria de Salud | Yes | Case reporting mandatory^1^ |
| **Namibia** | Ministry of Health and Social Services, Health Professions Council of Namibia | Yes^5^ | N/A |
| **Nepal** | Ministry of Health and Population | No^6,7^ | N/A |
| **Nicaragua** | Ministerio de Salud | No data | No data |
| **Panamá** | Ministerio de Salud | No data | No data |
| **Paraguay** | Ministerio de Salud Publica y Bienestar Social | No^1^ | N/A |
| **Philippines** | Philippine Regulatory Commission, Department of Health | Yes | Case reporting through national health information system^8^ |
| **Republic of Korea** | Ministry of Health and Welfare | Yes | Case reporting mandatory (since 1963);^1^ web-based surveillance system^9^ |
| **São Tomé and Príncipe** | Ministério da Saúde | No data | No data |
| **Saudi Arabia** | Ministry of Health | Yes | Case reporting mandatory (since 1990)^1^ |
| **Solomon Islands** | The Ministry of Health and Medical Services | No^10^ | N/A |
| **South Africa** | Department of Health | Yes | Weekly reports submitted to district offices^11,12^ |
| **Sri Lanka** | Ministry of Health and Private Health Services Regulatory Council | Yes | Case reporting mandatory (since 2008)^1^; toll-free line^13^ |
| **Swaziland** | Ministry of Health and Social Welfare and Swaziland Nursing Council^14^ | Yes | Reactive surveillance system using a toll-free hotline for all notifiable diseases^15^ |
| **Tajikistan** | Ministry of Health^16^ | Yes | Case reporting mandatory (since 2000)^1^ |
| **Thailand** | Ministry of Public Health and professional councils^17^ | No^18^ | N/A |
| **Turkey** | Ministry of Health^19^ | Yes | Case reporting mandatory (since 1930)^1,19^ |
| **Vanuatu** | Ministry of Health | No^20^ | N/A |
| **Vietnam** | Ministry of Health^21^ | No^22,23^ | N/A |

******No data* – no data found from publicly available references or retrieved from key informant interviews; *N/A* – not applicable.

**References:**

1 Global Malaria Programme. World malaria report 2014. 2014. http://www.who.int/malaria/publications/world_malaria_report_2014/wmr-2014-annexes.pdf?ua=1 (accessed Dec 8, 2014).

2 Ministry of Health and Social Assistance, Executive Commission for Health Sector Reform, Health Statistics Department, Regional Office of the Pan American Health Organization/World Health Organization. Health systems profile Dominican Republic: monitoring and analyzing health systems change, 3rd edn. Santo Domingo, Dominican Republic: PAHO/WHO, 2007.

3 Pan American Health Organization. Health systems profile Guatemala. 2007; published online Feb. http://new.paho.org/hq/dmdocuments/2010/Health_System_Profile-Guatemala_2007.pdf (accessed Dec 7, 2015).

4 Malaria Elimination Initiative. Eliminating malaria in Mayotte, France. 2015; published online March. http://globalhealthsciences.ucsf.edu/sites/default/files/content/ghg/country-briefings/Mayotte2015-FINAL.pdf (accessed Dec 7, 2015).

5 National Vector-borne Diseases Control Programme. Namibia malaria strategic plan 2010-2016. 2010; published online Nov.

6 World Health Organization Regional Office for South-East Asia. Nepal Malaria Programme Review. New Delhi, India, 2011.

7 Government of Nepal Ministry of Health and Population, Department of Health Services, Epidemiology & Disease Control Division. Nepal Malaria Strategic Plan 2011-2016. 2011; published online Dec.

8 Asia Pacific Malaria Elimination Network, Malaria Elimination Initiative. Strategy & intervention matrix: Philippines. 2011; published online Jan. http://apmen.org/storage/country-partner/APMEN Matrix Philippines 2011.pdf (accessed Nov 5, 2014).

9 Asia Pacific Malaria Elimination Network, Malaria Elimination Initiative. Strategy & intervention matrix: Republic of Korea. 2011; published online June. http://apmen.org/storage/country-partner/APMEN Matrix Republic of Korea 2011.pdf (accessed Nov 5, 2014).

10 Asia Pacific Malaria Elimination Network, Malaria Elimination Initiative. Strategy & intervention matrix: Solomon Islands. 2011; published online June. http://apmen.org/storage/country-partner/APMEN Matrix Solomon Islands 2011.pdf (accessed Nov 5, 2014).

11 Maharaj R, Raman J, Achour N, *et al.* Epidemiology of malaria in South Africa: from control to elimination. *S Afr Med J* 2013; **103**: 779–83.

12 Khosa E, Kuonza LR, Kruger P, Maimela E. Towards the elimination of malaria in South Africa: a review of surveillance data in Mutale Municipality, Limpopo Province, 2005 to 2010. *Malar J* 2013; **12**: 7.

13 Global Health Group, Anti Malaria Campaign Sri Lanka. Eliminating malaria in Sri Lanka. 2013; published online Aug. http://apmen.org/storage/country-briefings/Sri%20Lanka.pdf.

14 Ministry of Health. Swaziland nursing council. Gov. Kingd. Swazil. 2014. http://www.gov.sz/index.php?option=com_content&view=article&catid=73%3Ahealth&id=748%3Aswaziland-nursing-council&Itemid=286 (accessed Nov 18, 2014).

15 Cohen JM, Dlamini S, Novotny JM, Kandula D, Kunene S, Tatem AJ. Rapid case-based mapping of seasonal malaria transmission risk for strategic elimination planning in Swaziland. *Malar J* 2013; **12**: 61.

16 Khodjamurodov G, Rechel B. Tajikistan: health system review. *Health Syst Transit* 2010; **12**. http://www.hpi.sk/cdata/Documents/HIT/Tajikistan_2010.pdf (accessed Nov 5, 2014).

17 Teerawattananon Y, Tangcharoensathien V, Tantivess S, Mills A. Health sector regulation in Thailand: recent progress and the future agenda. *Health Policy* 2003; **63**: 323–38.

18 Asia Pacific Malaria Elimination Network, Malaria Elimination Initiative. Strategy & intervention matrix: Thailand. 2011; published online Sept. http://apmen.org/storage/country-partner/APMEN Matrix Thailand 2011.pdf (accessed Nov 17, 2014).

19 UCSF Global Health Group, Republic of Turkey Ministry of Health, World Health Organization. Eliminating malaria case-study 5: the long road to malaria elimination in Turkey. 2013. http://apps.who.int/iris/bitstream/10665/94961/1/9789241506403_eng.pdf (accessed Nov 17, 2014).

20 On ML, Bennett V, Whittaker M. Health information systems in the Pacific: a case study of Vanuatu. Herston Qld, Australia: School of Population Health University of Queensland, 2009.

21 Montagu D, Bloom A. The private sector and health services delivery in the EAP region: background report to UNICEF on the role and experiences of the private sector in provision of child health services. 2010. http://www.unicef.org/eapro/3_Market_decentralization_and_Health_Services_delivery.pdf (accessed Nov 17, 2014).

22 National Institute of Malariology, Parasitology and Entomology. National monitoring and evaluation plan for malaria control & elimination in Viet Nam for the period 2011-2020 (draft). 2011; published online March.

23 Asia Pacific Malaria Elimination Network, Malaria Elimination Initiative. Strategy & intervention matrix: Vietnam. 2014; published online March. http://apmen.org/storage/country-briefings/APMEN_Matrix_Vietnam_2013.pdf (accessed Nov 17, 2014).
