# Supplementary material for: Engaging the private sector in malaria surveillance: a review of strategies and recommendations for elimination settings
Source: Malar J. 2017 Jun 14;16:252. doi: 10.1186/s12936-017-1901-1 (PMC5471855; doi:10.1186/s12936-017-1901-1)
Supplement: Supplementary file 3 — Additional file 3. Case study narratives. [file 12936_2017_1901_MOESM3_ESM.docx]

**Additional File 3**. Country case study narratives

The following case studies examine the approaches used by countries in Southeast Asia and Southern Africa, represented by two malaria-eliminating (Swaziland and Vietnam) and four neighboring countries that either have sub-national elimination goals or are part of regional networks with elimination goals (Cambodia, Mozambique, Myanmar and Zambia), to engage the private sector in malaria surveillance and describe the successes and challenges of these strategies.

## Southeast Asia

## *Vietnam*

Vietnam has reduced its malaria burden significantly and consistently in the last decade. In 2013, there were estimated 35,406 cases of malaria in the country, which is less than half the number of cases in 2006 [1]. In contrast, malaria deaths went up to six in 2013 compared to zero deaths in the previous year [1].

While the public sector provides most institutional and specialty care in the country (e.g., birth delivery services) [2,3], the private sector delivers over 60% of all outpatient care, particularly for young children [4–6]. In some areas, the number of private providers, including pharmacies and drug vendors, is double that of public providers and facilities [7]. For diseases such as tuberculosis (TB), as many as 80% of individuals use the private sector for care [8], and among patients who are diagnosed with TB in the public sector, approximately 50% of them first sought care from a private provider [9,10].

The Malaria Information System or other parallel reporting systems in Vietnam currently does not collect data from the private sector [11,12], thus it is unclear what proportion of malaria cases present to private facilities. Despite the poor integration of the private sector in disease surveillance, Vietnam has a number of successful public-private partnerships (PPPs) to improve access to essential health services and commodities that may serve as a good foundation for better private sector engagement for malaria. For example, Vietnam has addressed a projected $45 million contraceptive budget shortfall by embracing a total market approach for contraceptives [13]. This entails the national government working closely with private sector stakeholders to understand market demand and manage targeting of resources and supplies. Additionally, the creation of a multi-sectoral group, which includes the commercial sector, to oversee the monitoring and evaluation of the contraceptive market will be critical to meet changing market demands [13]. Demonstrating success in meeting demand across the public and private sectors would be of particular interest for malaria programs.

Vietnam also has implemented PPPs for TB care, which has been shown to improve case detection of new smear-positive cases but has met with mixed success in terms of treatment outcomes [14–16].

Population Services International (PSI) Vietnam is using social franchising strategies to improve the access and quality of health services, as well as reporting. Under the brand name “Good Health, Great Life,” 150 private clinics are franchised in four provinces and have a contractual agreement to uphold franchise standards, including maintaining national critical health services, reporting monthly results and participating in trainings and meetings [17]. Private clinics benefit by being able to promote the quality brand and receiving additional trainings. The government benefits by having private providers comply with national guidelines for health services and reporting. While none of the social franchise programs in Vietnam currently provide malaria testing and treatment, organizations like PSI Vietnam are consulting with the National Malaria Control Program (NMCP) to understand how they can apply their core competencies in behavior change communication (BCC), product marketing and social franchising to improve malaria control efforts, including improved surveillance within the private sector.

***Cambodia***

Cambodia has made significant gains in reducing malaria morbidity and mortality. In 2013, 24,130 presumed and confirmed malaria cases were reported, along with 12 malaria deaths [1]. Compared to figures from 2012, malaria cases in Cambodia have been reduced by more than half and malaria deaths reduced by more than two thirds.

The primary source of healthcare in Cambodia is the private sector, constituting an estimated 80% of all health treatment. Private pharmacies are often the first point of contact for the majority of ill individuals [18,19]. Approximately 70% of malaria patients reported seeking treatment in the private sector, and approximately 75% of antimalarials in the country are provided by the private sector [20]. The private sector, which is largely unregulated in Cambodia, includes both registered providers (pharmacists, doctors and nurses) and unregistered providers (drug sellers and itinerant vendors) in addition to small and large private companies such as agriculture and construction enterprises that provide health services to their workers.

Local village vendors are the most common source of malaria drugs, followed by private health workers and public health facilities. The National Center for Parasitology, Entomology and Malaria Control (CNM), has increased efforts to regulate and monitor private drug sellers in order to contain *Plasmodium falciparum* artemisinin resistance, which was first documented in the country [21]. As part of the regulation efforts, CNM has created a bureau dedicated to ensuring that providers are registered and to increasing enforcement of non-registered providers selling medicines including artemisinin monotherapies (AMTs). The Cambodian government, with support from the U.S. government, has been effective in regulating illegal outlets selling antimalarials by closing the number of outlets by 65% between 2009 and 2010 from 1,081 to 379, respectively [22]. Additionally, the Department of Drug and Food within the Ministry of Health (MOH) has drug inspection police who monitor private pharmacies on a regular basis and sample malaria drugs in both the public and private sectors to ensure quality of antimalarials and prevent the sale of AMTs [23].

Evidence generated by ACTwatch outlet surveys has supported decision-making in Cambodia with regards to drug policy and strategies for drug implementation and scale-up, including engagement with the private sector [24]. For example, in an effort to shift the private sector towards more formal regulated outlets, the proportion of unregulated private sector antimalarial-stocking outlets was reduced from 70% to 43% between 2009 and 2013 [25]. This experience was useful for Cambodia to understand private sector market composition over time, and can be applied in other settings to monitor policy change effects.

Cambodia was the first country to pilot and scale up the provision of subsidized artemisinin combination therapies (ACTs) in the private sector. PSI has provided malaria services through the private sector in Cambodia since 2003. This includes distributing and subsidizing malaria rapid diagnostic tests (RDTs) and Malarine, a prepackaged ACT (artesunate plus mefloquine), which accounts for as much as 48% of the market share for antimalarial drugs in some regions [25,26]. This experience has demonstrated that routine interaction and trainings with the private providers must be included to develop relationships and assure provider use. Despite this success, a challenge will be to identify the appropriate financial incentives and training program to achieve the desired provider and consumer behaviors for diagnosis and quality treatment, particularly in the face of a declining malaria caseload [27].

In Cambodia, as elsewhere, tracking patients that visit private providers is difficult, and many are lost to follow up. To address this, a short message service (SMS) alert system was implemented for village malaria workers, CNM and its partners including Malaria Consortium and other groups. This public-private mix (PPM) project aims to train private providers on appropriate diagnosis, treatment and referral procedures [28]. Targeting areas with the highest risk of drug resistance, the pilot referral system was implemented with more than 100 private providers to give a more accurate picture of the malaria burden and avoid duplication of registered cases in the public sector. If a private provider at a private clinic suspects a malaria case, he/she can SMS message the referral slip number and patient’s phone number to the database. When the patient arrives at the public clinic, their referral information has already been captured in the database and can be matched to the original private clinic referral. This allows the CNM to account for patients at public facilities by tracking referrals to estimate caseload and track commodities by matching diagnostics to treatment. Early results indicate that 65% (104 of 160) of referrals reached the public sector [29]. Only non-financial incentives such as trainings and completion certificates were provided to the private providers and may have contributed to fewer referrals overall based on expected caseload. The SMS referral system is an innovative way to enable private providers to participate in patient care while adhering to national guidelines. Partnering with telecommunication companies was a key component to ensure that all SMS messages were sent free of charge. Despite the success of this system, challenges remain, including not knowing whether all patients visiting the private providers and needing to be referred actually have been. Additionally, the referral system does not capture patients visiting providers that are not in the referral network.

To strengthen the linkages between the private and public health sectors and thereby improve health outcomes, the CNM commissioned PATH to develop a PPM model in two Cambodian provinces [30]. The PATH PPM aimed to build partnerships and linkages between the public and private sectors to improve coordination and reporting. The project implemented regularly scheduled meetings between these two stakeholders to discuss the benefits and challenges experienced by private providers, particularly those are unregistered and informal which provide malaria services. Additionally, more than 250 private providers were trained on appropriate malaria diagnosis, treatment and referrals. Through this collaboration, trust between the public and private sectors has been established to facilitate improved communication.

***Myanmar***

More than 50% of malaria cases and about 75% of malaria deaths in the Greater Mekong Subregion occur in Myanmar [31,32]. In 2013, 333,871 presumed and confirmed malaria cases were reported in the country, along with 236 malaria deaths [1].

It is estimated that roughly 80% of all care in Myanmar is provided by private providers which include physicians, midwives, traditional healers and health facilities run by faith-based organizations and nongovernmental organizations (NGOs) [33,34]. While the public system in Myanmar is highly structured and provides most of the specialty and inpatient care, private providers are the largest source of ambulatory care in the country and includes reproductive health services and infectious disease diagnostics and treatment [33–36]. While there have been conflicting findings about the relative contributions of the public and private sectors in overall malaria testing and treatment in Myanmar, an ACTwatch survey conducted in 2012 concluded that private clinics and retail outlets are a more common source of antimalarials than the public sector [37,38].

Myanmar has one of the most active social franchising programs globally through the work of PSI, who has emerged as one of the largest providers of healthcare services and products in the country [39]. PSI manages two private provider networks in Myanmar that provide primary care, family planning and reproductive health commodities and services and malaria diagnostic and treatment services, among others [40]. Sun Quality Health (SQH) is composed of physicians and advanced health professionals and serves primarily urban and peri-urban populations [41,42]. Sun Primary Health (SPH) is composed of lower level health professionals such as midwives [41]. In 2013, over 3,300 private providers were registered in these two networks [43]. SQH and SPH contribute significantly to malaria diagnostics and treatment in Myanmar. In 2013 alone, these two networks tested approximately 350,000 suspected malaria cases and treated approximately 50,000 confirmed cases [39].

As part of the Myanmar Artemisinin Resistance Containment (MARC) project funded by the Three Diseases Fund and led by the MOH, the Myanmar Medical Association (MMA) has procured RDTs and rolled out their use, conducted mobile visits to reach at-risk populations, trained village health workers who conduct community-based malaria control and recruited general practitioners to several townships previously without providers [44–46]. MMA, which boasts a membership of over 17,000 registered medical providers, also provides training and refresher programs to its members on current malaria treatment guidelines [47]. MMA is also working with Malaria Consortium and other partners to roll out integrated vector management and BCC interventions in pilot communities [48].

A key component of MARC is the Artemisinin Monotherapy Replacement (AMTR) project co-funded by the Bill & Melinda Gates Foundation and the U.K. Department for International Development and implemented by PSI. Initiated in 2011, AMTR aimed to replace oral AMT sold in the private sector with quality-assured ACT (QAACT) [49,50]. This was achieved by working with two licensed drug distribution companies in Myanmar, AA Pharmaceuticals and PolyGold, to halt the importation of AMT and implement a nationwide ban on AMT sales. These distributors were then given access to subsidized QAACT (artemether lumefantrine), and as of November 2014, over 1.3 million QAACTs have been sold to these two distributors (White *et al.* 2014). PSI also conducted medical detailing to private pharmacies, itinerant drug vendors and general retailers by sending promoters to explain the importance of QAACTs and encourage its use in fever case management. Collectively these drug sellers provide 21% of initial fever treatment, 15.7% of treatment for malaria diagnoses and 13% of all antimalarials in Myanmar [38]. Through a public communication campaign, consumers were also educated about QAACTs to increase demand. The campaign focused on the pandoma logo, the quality-assurance seal used on ACTs approved by the WHO and the government. As of June 2013, AMTR has increased the availability of ACTs to 63% from 27% in 2012 among private drug outlets [51]. Additionally, the market share of ACTs has increased relative to AMTs. After nine months of AMTR implementation, over 73% of all antimalarial drugs sold in the pilot townships were ACTs [51]. As part of the second phase of the project, PSI is planning to introduce RDTs to the private sector and improve the rational use of QAACTs [52].

Myanmar is also another example of a country with active engagement with private industries such as palm oil and rubber plantations, prawn farms, gold panning and gem mining, all of which employ a significant number of mobile laborers, a group that is at higher risk of contracting malaria [36,53]. The NMCP assists these companies and provides technical guidance on developing their diagnostic and treatment services, BCC interventions and prevention strategies for malaria among their workers. In return, the NMCP seeks malaria surveillance data from these private companies, although it remains unclear if case reporting already takes place on a regular basis [36].

Challenges with fully engaging the private sector remain, however. NGOs and private providers are not formally integrated in the national health management information system (HMIS), although efforts to change this are underway [36]. In 2011, a new case reporting system was adopted by the NMCP which utilized a new form that NGOs were mandated to use [54]. The NMCP is now developing guidelines and tools, including mobile health solutions, to standardize case data reporting among public and private health workers at every township and to facilitate rapid and complete reporting [55]. PSI has also mapped over 10,000 drug outlets in pilot townships where it aims to roll out the use of District Health Information Software 2 through mobile technology for routine data collection, entry and reporting. This project will be rolled out in select townships in eastern Myanmar in 2015 and scaled up the following year.

Southern Africa

## *Swaziland*

Malaria in the Kingdom of Swaziland has declined significantly in the past decade, from 4,005 cases in 2001 to 369 in 2012 [56,57]. Only the eastern half of the country is receptive to malaria, and the NMCP attempts to follow-up every case detected in the receptive area and conduct reactive case detection.

In Swaziland, the private sector consists of hospitals, private, industry and NGO clinics, and pharmacies and comprises approximately 10–15% of Swaziland’s health sector. One hundred and twenty-eight private facilities were identified in a service availability mapping evaluation in 2013, and 93 of these facilities were identified as offering any malaria services [58].

The majority of malaria patients in Swaziland use public health facilities where they receive an RDT and are prescribed ACTs. Following a positive RDT, the MOH mandates that all health providers, both public and private, report the case to the immediate disease notification system (IDNS) by phone within 24 hours, a system developed and managed by the NMCP with support from the Clinton Health Access Initiative (CHAI) [56]. In 2013, the NMCP and CHAI worked together to assess capacity, barriers and incentives for engaging private sector health providers in malaria case management and reporting into the IDNS [58]. Through this process the NMCP learned that while the private sector is eager to participate in more effective surveillance for malaria elimination, the lack of knowledge about how to effectively engage with the MOH and report in a systematic manner is an obstacle to effective inclusion. While the majority of providers reported to the monthly HMIS system, only half reported to the IDNS. Most private sector representatives were not familiar with the IDNS, and many were unaware that the purpose of reporting a case is to trigger a community-level investigation. As a result, the NMCP now visits private health facilities monthly to offer one-hour malaria trainings that better fit the schedules of those facilities [58], and members of the private sector are instructed to report malaria cases to the IDNS in the same manner as public health facilities. This includes sugar plantations, which now report cases to the IDNS, and receive trainings on malaria elimination strategies, national case management and surveillance guidelines. In addition, as a result of discussions between the NMCP and private sector representatives, the NMCP provides RDTs and ACTs to private facilities free of charge and NMCP program officers make regular visits to private providers to address difficulties with reporting.

In the Swaziland context, it has been essential for the MOH and private sector to sit together, discuss their needs and capacities and for the MOH to change the way they do some things (e.g., give trainings) rather than mandate that the private sector participate in the existing system. Challenges remain in that a bill governing private health facilities does not exist, so that the MOH has limited authority to sanction facilities that refuse to report, and there is no method in place to incentivize reporting. Additionally, there are no specific guidelines for engaging the private sector, and decisions are made according to the needs of each health facility.

***Mozambique***

In 2013, there were over 3.9 million presumed and confirmed malaria cases and over 2,900 malaria deaths in Mozambique. Compared to 2007, Mozambique has reduced its malaria cases by almost 40% while malaria deaths have increased by almost 70% [1].

The private healthcare sector in Mozambique is relatively small, primarily comprised of health facilities operated by private industry and private pharmacies that sell RDTs but not ACTs. Many privately owned health facilities are funded by donors who choose to channel funding into NGO-run health facilities rather than the public sector [59]. Currently, there are no regulations that mandate private sector participation in the national surveillance system, although malaria is a reportable disease. In addition, there are no existing channels for private sector health facilities to report malaria data routinely.

Private industry healthcare is the most successful example of private sector activities in Mozambique. Some coal mining and natural gas companies provide healthcare to their workers, including malaria prevention, testing and treatment. As they have a vested interest in keeping their employees healthy, these companies engage in malaria surveillance and treatment—including tracking malaria commodities and cases—and conduct parasitemia or baseline studies, the results of which have been shared with the Ministério da Saúde (MISAU, Mozambique’s MOH).

A significant challenge to engaging the private sector in Mozambique as shared by one key informant is the political and financial influence of private companies within the country, which may make the government hesitant to ask for or mandate reporting. Though uncommon, another challenge to engaging the private sector is the conflict that sometimes exist between the aims of the private company implementing corporate social responsibility projects and the aims of MISAU’s strategic plan for malaria [59].

While Mozambique is at the beginning stages of engaging the private sector in malaria surveillance, the potential inclusion of this work in the national strategic plan is encouraging. Given the challenges faced by Mozambique’s public health facilities, including frequent commodity stockouts and lack of electricity and running water, involvement of the private sector could significantly increase access to quality health services [60,61]. Additionally, one company’s partnership with an NGO currently working on malaria in the country is a promising example of engaging in corporate social responsibility projects for malaria by relying on NGOs with local experience and existing relationships with the NMCP.

*Zambia*
Zambia remains a high malaria burden country but has the ambitious goal to achieve elimination by 2030. In 2013, over 5.4 million cases of presumed and confirmed malaria cases were reported, along with 3,548 malaria related deaths [1].

Zambia’s healthcare system is comprised of a decentralized public health system and the private sector. The public health system is managed by the MOH and serves as the main provider of healthcare services. A significant number of public health facilities, however, are run by Churches Health Association of Zambia (CHAZ), an association formed in 1970 by 16 Catholic and Protestant groups [62]. CHAZ is funded largely by the MOH and provides approximately 35% of healthcare services in Zambia [62,63].

The private sector—comprised of for-profit hospitals and clinics, employer-owned and NGO-based health facilities, pharmacies, drug stores and general retailers or shops—is a less significant source of healthcare in Zambia. In 2009, there were only 432 registered private medical providers in the country and only 14% of the 1,882 formal healthcare facilities were privately owned and operated in 2010 [64,65]. Private providers however deliver antenatal, child and maternal care, HIV/AIDS prevention and treatment, contraception commodities and services and immunizations to many Zambians [66–68], and evidence suggests that compared to the public sector, the private sector provides equal or greater quality care [69].

The latest ACTwatch survey found that across all age groups, the majority of Zambians first utilize the public sector for advice or treatment of fevers [70], and it is the primary source of diagnosis for fevers and antimalarials. Less than a quarter of Zambians seek care for fever in the private sector, as a result they are a less significant source of fever diagnoses and antimalarials [70].

Despite the less central role that the private sector plays in the Zambian health system, several efforts have been made to integrate them into national disease programs or expand their reach in the communities they operate. For example, the Zambia Access to ACTs Initiative (ZAAI) funded by U.K. Department of International Development and the World Bank was implemented in 2011 to explore the regulation of private drug shops in rural and remote areas [71]. Subsidized ACTs and RDTs were sold to wholesalers and distributed to accredited private drug sellers in pilot districts through normal distribution channels. After the 10-month pilot study, 50 health shops were accredited, more than 22,430 people were tested for malaria and over 7,764 people were treated with effective antimalarials [72]. Access to and affordability of ACTs in the private sector increased, as well as the diagnostic capacity of drug sellers, and the use of ineffective antimalarials decreased. Additionally, it was found that care-seeking for children in public and private facilities increased among pilot districts compared to control districts [73].

Because the provision of subsidized commodities was tied to record keeping, most drug shops maintained records of the number of ACTs they dispensed and the results of the RDTs used. These records were accessible to district health officers, but it is unclear if they routinely collected or used this data. Similar drug shop accreditation programs such as the accredited drug dispensing outlets in Tanzania also incorporated guidelines for improved record keeping on drug consumption quantities, patient-related data, adverse drug reactions and product losses due to drug expiry [74–76]. These efforts were shown to be effective although the data were never communicated to any health authority [77].

Recognizing both the success of ZAAI and the limitations of current regulations, the Pharmaceutical Regulatory Authority (PRA) in Zambia proposed amendments to the 2004 pharmaceutical law [73]. In 2013, the Medicines and Allied Substances Act was passed by the Zambian Parliament that allows the PRA (which has been renamed to the Zambian Medicines Regulatory Authority) to register and issue licenses to health shops, allowing these sellers to stock and sell drugs from a prescribed list including ACTs [78]. This change will help increase access to quality-assured RDTs and ACTs in areas where health shops operate but will pose a new challenge for malaria surveillance.

Private companies have historically played a large role in malaria control. A recent assessment of the impact made by the prevention and control programs of three private companies in Zambia (i.e., Zambia Sugar Plc, Mopani Copper Mines Plc and Konkola Copper Mines Plc) which implemented indoor residual spraying, distributed insecticide treated nets, and conducted on-site case management, found that from 2000-2009, annual malaria cases decreased by 94% and over 108,000 malaria episodes and 300 deaths were averted in the areas they work [79]. In addition, malaria-related workdays lost and healthcare spending decreased by 94% and 76% respectively. To build on the successes of these companies, engagement with the Zambia Association of Chambers of Commerce and Industry is underway to expand malaria control efforts to other sectors [80]. However, experts caution that financial support from private companies for malaria prevention and treatment activities may wane once malaria becomes an uncommon disease [81].

Integrating private sector actors into malaria surveillance systems remains a challenge. The national HMIS, as well as the newer District Health Information System, routinely receive data from public facilities and large private hospitals but excludes many smaller private clinics and health facilities, pharmacies and shops [80,82]. Generally, private sector reporting in Zambia is poor and there is currently no legislation that requires private providers to report case data to the national government [82].

**Acronyms**

ACT – Artemisinin combination therapy

AMT – Atemisinin monotherapies

AMTR – Artemisinin Monotherapy Replacement

BCC – Behavior change communication

CHAI – Clinton Health Access Initiative

CHAZ – Churches Health Association of Zambia

CNM – National Center for Parasitology, Entomology and Malaria Control

HMIS – Health management information system

IDNS – Immediate disease notification system

MARC – Myanmar Artemisinin Resistance Containment

MISAU – Ministério da Saúde

MMA – Myanmar Medical Association

MOH – Ministry of Health

NGO – Nongovernmental organization

NMCP – National Malaria Control Program

PRA – Pharmaceutical Regulatory Authority

PPM – Public-private mix

PPP – Public-private partnership

PSI – Population Services International

QAACT – Quality-assured artemisinin combination therapy

RDT – Rapid diagnostic test

SMS – Short message service

SQH – Sun Quality Health

SPH – Sun Primary Health

TB – Tuberculosis

ZAAI – Zambia Access to ACTs Initiative

**References**

1. Global Malaria Programme. World malaria report 2014 [Internet]. World Health Organization; 2014 [cited 2014 Dec 8]. Available from: http://www.who.int/malaria/publications/world_malaria_report_2014/wmr-2014-annexes.pdf?ua=1

2. Do M. Utilization of skilled birth attendants in public and private sectors in Vietnam. J. Biosoc. Sci. 2009;41:289.

3. Ngo AD, Hill PS. The use of reproductive healthcare at commune health stations in a changing health system in Vietnam. BMC Health Serv. Res. 2011;11:237.

4. Ha NTH, Berman P, Larsen U. Household utilization and expenditure on private and public health services in Vietnam. Health Policy Plan. 2002;17:61–70.

5. Thuan NTB, Lofgren C, Lindholm L, Chuc NTK. Choice of healthcare provider following reform in Vietnam. BMC Health Serv. Res. 2008;8:162.

6. Anh Duc H, Sabin LL, Cuong LQ, Duc Thien D, Feeley R. Potential collaboration with the private sector for the provision of ambulatory care in the Mekong region, Vietnam. Glob. Health Action. 2012;5:10126.

7. Tuan T, Dung VTM, Neu I, Dibley MJ. Comparative quality of private and public health services in rural Vietnam. Health Policy Plan. 2005;20:319–27.

8. PSI/Vietnam Research and Metrics. Tuberculosis knowledge and care seeking behavior among people with tuberculosis symptoms: Vietnam report 2014. PSI/Vietnam; 2014.

9. Lönnroth K, Linh PD, Diwan VK, others. Utilization of private and public health-care providers for tuberculosis symptoms in Ho Chi Minh City, Vietnam. Health Policy Plan. 2001;16:47–54.

10. PSI/Vietnam. Tuberculosis (TB) health-seeking behavior: qualitative insights from Dong Thap and Dong Nai provinces of Vietnam. PSI/Vietnam; 2012.

11. Erhart A, Thang ND, Xa NX, Thieu NQ, Hung LX, Hung NQ, et al. Accuracy of the health information system on malaria surveillance in Vietnam. Trans. R. Soc. Trop. Med. Hyg. 2007;101:216–25.

12. National Institute of Malariology, Parasitology and Entomology. National monitoring and evaluation plan for malaria control & elimination in Viet Nam for the period 2011-2020. Vietnam Ministry of Health; 2011.

13. Drake JK. Developing a total market plan for family planning in Vietnam. Program for Appropriate Technology in Health (PATH); 2011.

14. PATH. Public-private mix for tuberculosis control: lessons learned from the USAID-supported project in Vietnam. USAID; 2013.

15. Quy HT, Lan NTN, Lönnroth K, Buu TN, Dieu TTN, Hai LT. Public-private mix for improved TB control in Ho Chi Minh City, Vietnam: an assessment of its impact on case detection. Int. J. Tuberc. Lung Dis. 2003;7:464–471.

16. Quy HT, Lönnroth K, Lan NTN, Buu TN. Treatment results among tuberculosis patients treated by private lung specialists involved in a public-private mix project in Vietnam. Int. J. Tuberc. Lung Dis. 2003;7:1139–1146.

17. Viswanathan R, Schatzkin E, Sprockett A. Clinical social franchising compendium: an annual survey of programs: findings from 2013 [Internet]. UCSF Global Health Group; 2014. Available from: http://sf4health.org/sites/sf4health.org/files/wysiwyg/Social-Franchising-Compendium-2014_0.pdf

18. Montagu D. Large-scale malaria treatment in the private sector: a case study of the Cambodian experience. The Global Health Group, University of California, San Francisco; 2010.

19. Patouillard E, Hanson K, Kleinschmidt I, Palafox B, Tougher S, Pok S, et al. Determinants of price setting decisions on anti-malarial drugs at retail shops in Cambodia. Malar. J. 2015;14:244.

20. Mellor S. Moving towards malaria elimination: developing innovative tools for malaria surveillance in Cambodia. Malaria Consotrium; 2013 Dec.

21. National Malaria Control Centre. The national strategic plan for elimination of malaria in the Kingdom of Cambodia 2011-2025 [Internet]. Royal Government of Cambodia; 2011. Available from: http://www.cnm.gov.kh/userfiles/file/N Strategy Plan/National Strtegyin English.pdf

22. Moszynski P. Cambodia cracks down on illegal drug vendors in bid to counter antimalarial resistance. BMJ. 2010;340:c2622.

23. Chuor CM. Benefits to national malaria programs from regional support: the Cambodia case [Internet]. Asia Pacific Leaders Malaria Alliance; 2014 [cited 2014 Nov 14]. Available from: http://aplma.org/upload/resource/paper/Benefits to National Malaria Programs from Regional Support The Cambodia case.pdf

24. ACTwatch. Providing malaria market evidence through the ACTwatch project [Internet]. Population Services International; 2013 [cited 2014 Dec 19]. Available from: http://www.actwatch.info/sites/default/files/content/publications/attachments/PSI Brochure PRESS v2.pdf

25. ACTwatch. Cambodia 2013 outlet survey results [Internet]. Population Services International; 2013. Available from: http://www.actwatch.info/sites/default/files/content/publications/attachments/Cambodia_OS_2013_Brief.pdf

26. Littrell M, Gatakaa H, Phok S, Allen H, Yeung S, Chuor CM, et al. Case management of malaria fever in Cambodia: results from national anti-malarial outlet and household surveys. Malar. J. 2011;10:10–1186.

27. Yeung S, Patouillard E, Allen H, Socheat D. Socially-marketed rapid diagnostic tests and ACT in the private sector: ten years of experience in Cambodia. Malar. J. 2011;10:243.

28. Malaria Consortium. Private sector SMS referral system pilot [Internet]. Cambodia; 2013. Available from: http://www.malariaconsortium.org/media-downloads/229/Private sector SMS referral system pilot

29. Brenden N, Jitthai N. Evaluation of the malaria control in Cambodia project: final report [Internet]. USAID; 2012 [cited 2014 Nov 14]. Available from: http://pdf.usaid.gov/pdf_docs/PDACU493.pdf

30. PATH. Piloting a malaria public-private mix model in Cambodia [Internet]. PATH; 2011 [cited 2014 Aug 14]. Available from: http://www.path.org/publications/files/CP_cambodia_malaria_ppm.pdf

31. Cui L, Yan G, Sattabongkot J, Cao Y, Chen B, Chen X, et al. Malaria in the Greater Mekong Subregion: heterogeneity and complexity. Acta Trop. 2012;121:227–39.

32. Roll Back Malaria Partnership, Asia Pacific Leaders Malaria Alliance, GBC Health. Opportunities for corporate sector engagement in malaria control in the Asia-Pacific [Internet]. 2014 [cited 2014 Dec 8]. Available from: http://www.rbm.who.int/globaladvocacy/pr2014-09-29.html

33. Risso-Gill I, McKee M, Coker R, Piot P, Legido-Quigley H. Health system strengthening in Myanmar during political reforms: perspectives from international agencies. Health Policy Plan. 2014;29:466–74.

34. Myanmar Ministry of Health. Health in Myanmar 2012 [Internet]. Myanmar Ministry of Health; 2012 [cited 2014 Aug 15]. Available from: http://www.searo.who.int/myanmar/documents/healthinmyanmar2012/en/

35. Lynn KS. Healthcare in Myanmar [Internet]. Ipsos Business Consulting; 2013. Available from: http://www.ipsosconsulting.com/pdf/Research-Note-Healthcare-in-Myanmar.pdf

36. World Health Organization. Myanmar external evaluation of the national malaria control programme 30 July-9 August 2012 [Internet]. World Health Organization; 2012. Available from: http://apps.who.int/iris/bitstream/10665/94362/1/2012-10SEA_CDS_Myanmar_Evaluation%20of%20National%20Malaria%20Control%20Prog.2012.pdf

37. Xu J-W, Xu Q-Z, Liu H, Zeng Y-R. Malaria treatment-seeking behaviour and related factors of Wa ethnic minority in Myanmar: a cross sectional study. Malar. J. 2012;11:417.

38. PSI Myanmar. Household survey: baseline. The Republic of the Union of Myanmar 2012 AMTR survey report [Internet]. Population Services International; 2013 [cited 2014 Oct 15]. Available from: http://www.actwatch.info/sites/default/files/content/publications/attachments/Myanmar Baseline Household Survey Report.pdf

39. PSI. Myanmar [Internet]. PSI. 2010 [cited 2014 Aug 15]. Available from: http://www.psi.org/myanmar

40. Schlein K, Drasser K, Montagu D. Clinical social franchising case study series: Sun Quality Health, Population Services International/Myanmar [Internet]. The Global Health Group, University of California, San Francisco; 2010. Available from: http://www.sf4health.org/sites/sf4health.org/files/reports/Case-Study-Myanmar.pdf

41. Bishai D, LeFevre A, Theuss M, Boxshall M, Hetherington JD, Zaw M, et al. The cost of service quality improvements: tracking the flow of funds in social franchise networks in Myanmar. Cost Eff. Resour. Alloc. 2013;11:14.

42. O’Connell K, Hom M, Aung T, Theuss M, Huntington D. Using and Joining a Franchised Private Sector Provider Network in Myanmar. Noor AM, editor. PLoS ONE. 2011;6:e28364.

43. Montagu D, Longfield K, Briegleb C, Aung T. Private sector healthcare in Myanmar: evidence from the “Sun” social franchise [Internet]. PSI and UCSF Global Health Group; 2013. Available from: http://www.psi.org/sites/default/files/publication_files/Private_Sector_Healthcare_Myanmar.pdf

44. 3DF. MARC Implementing Partners and their projects [Internet]. Three Dis. Fund. 2012 [cited 2014 Oct 17]. Available from: http://www.3dfund.org/index.php?option=com_content&view=article&id=139&Itemid=112

45. SEARO Country Office for Myanmar. Improving quality of management of malaria by the private general practitioners [Internet]. Reg. Off. South-East Asia. 2014 [cited 2014 Oct 17]. Available from: http://www.searo.who.int/myanmar/areas/malariaqualitymgnt/en/

46. Myanmar Medical Association. MMA malaria project at a glance [Internet]. Myanmar Medical Association; 2014 [cited 2014 Oct 15]. Available from: http://pr.org.mm/wp-content/uploads/2014/04/myanmar_medical_association.pdf

47. Kyaw S. Country reports: Myanmar Medical Association. Jpn. Med. Assoc. J. 2012;55:459–62.

48. Malaria Consortium. Project brief: regional integrated vector management project [Internet]. Malaria Consortium; 2013 [cited 2014 Oct 15]. Available from: http://www.malariaconsortium.org/media-downloads/217/Regional Integrated Vector Management Project

49. U.K. Department for International Development. Annual review: replacement of malaria monotherapy drugs in the private sector to support containment of drug resistant malaria in eastern Burma (AMTR) [Internet]. DFID; 2012 [cited 2014 Jan 22]. Available from: http://iati.dfid.gov.uk/iati_documents/3776759.docx

50. Khin HSS, Chen I, White C, Sudhinaraset M, McFarland W, Littrell M, et al. Availability and quality of anti-malarials among private sector outlets in Myanmar in 2012: results from a large, community-based, cross-sectional survey before a large-scale intervention. Malar. J. [Internet]. 2015 [cited 2015 Nov 23];14. Available from: http://www.malariajournal.com/content/14/1/269

51. White C, Allen H, Littrell, Megan, Spiers A. Market interventions to improve access to quality medicines and diagnostics: generating evidence for malaria medicine policy in the Asia-Pacific region [Internet]. Asia Pacific Leaders Malaria Alliance; 2014 [cited 2014 Jan 22]. Available from: http://aplma.org/upload/resource/Papers/item 4 market interventions to improve access to quality medicines and diagnostics_final.pdf

52. Sudhinaraset M, Briegleb C, Aung M, Khin H, Aung T. Motivation and challenges for use of malaria rapid diagnostic tests among informal providers in Myanmar: a qualitative study. Malar. J. 2015;14:61.

53. Nyunt MH, Aye KM, Kyaw MP, Kyaw TT, Hlaing T, Oo K, et al. Challenges in universal coverage and utilization of insecticide-treated bed nets in migrant plantation workers in Myanmar. Malar. J. 2014;13:211.

54. Cox J. Evaluation of community- and health facility-based systems for the surveillance of cases of day-3 positive Plasmodium falciparum in Cambodia [Internet]. Malaria Consortium; 2011. Available from: http://www.malariaconsortium.org/resources/publications/165/evaluation-of-community-and-health-facility-based-systems-for-the-surveillance-of-cases-of-day-3-positive-plasmodium-falciparum-in-cambodia

55. Cox J, Mellor S. Consultancy report: assessment of malaria surveillance in Myanmar [Internet]. Malaria Consortium; 2013. Available from: http://www.malariaconsortium.org/media-downloads/325/Assessment of Malaria Surveillance in Myanmar

56. Kunene S, Phillips AA, Gosling RD, Kandula D, Novotny JM. A national policy for malaria elimination in Swaziland: a first for sub-Saharan Africa. Malar. J. 2011;10:313.

57. The Roll Back Malaria Partnership, Adams H, Kunene S, Mouzin E, Novotny J, Phillips AA, et al. Focus on Swaziland. Geneva, Switzerland: Roll Back Malaria Partnership Secretariat; 2012.

58. National Malaria Control Programme, Clinton Health Access Initiative. An assessment of capacity, barriers, and incentives for engaging private health care providers in malaria elimination efforts in Swaziland. Kingdom of Swaziland Ministry of Health; 2013.

59. Mussa AH, Pfeiffer J, Gloyd SS, Sherr K. Vertical funding, non-governmental organizations, and health system strengthening: perspectives of public sector workers in Mozambique. Hum. Resour. Health. 2013;11.

60. World Health Organization. Mozambique Health Profile [Internet]. 2012. Available from: http://www.who.int/gho/countries/moz.pdf

61. The United States Global Health Initiative. Mozambique Strategy: 2011 - 2015 [Internet]. 2011. Available from: http://www.ghi.gov/whereWeWork/docs/MozambiqueStrategy.pdf

62. Mudenda D, Mapoma CC, Chitah B, Chompolola A, Wake W. Provider purchasing and contracting for health services: the case of Zambia [Internet]. The Rockefeller Foundation and Results for Development Institute; 2008 [cited 2014 Sep 19]. Available from: http://saipar.org:8080/eprc/handle/123456789/223

63. Churches Health Association of Zambia. About us [Internet]. Churches Health Assoc. Zamb. 2013 [cited 2014 Oct 16]. Available from: http://www.chaz.org.zm/?q=about_us

64. McKeon K, Musona D. Financing and business development needs of private health care providers in Zambia - market research report [Internet]. Banking on Health Project, Abt Associates, Inc.; 2009 [cited 2014 Oct 15]. Available from: http://www.banyanglobal.com/pdf/Market_Research_Report-Financing_and_Business_Development_Needs_of_Private_Health_Care_Providers_in_Zambia.pdf

65. President’s Malaria Initiative. President’s Malaria Initiative Zambia: malaria operational plan FY 2014 [Internet]. USAID; 2014 [cited 2014 Oct 15]. Available from: http://www.pmi.gov/docs/default-source/default-document-library/malaria-operational-plans/fy14/zambia_mop_fy14.pdf?sfvrsn=8

66. Central Statistical Office, Ministry of Health, Tropical Diseases Research Centre, University of Zambia, Macro International Inc. Zambia demographic and health survey 2007 [Internet]. CSO and Macro International Inc.; 2009. Available from: http://dhsprogram.com/pubs/pdf/FR211/FR211[revised-05-12-2009].pdf

67. Sipilanyambe N, Simon JL, Chanda P, Olumese P, Snow RW, Hamer DH. From chloroquine to artemether-lumefantrine: the process of drug policy change in Zambia. Malar. J. 2008;7:25.

68. Berman P, Nwuke K, Rannan-Eliya R, Mwanza A. Zambia: non-governmental health care provision [Internet]. Harvard School of Public Health; 1995 [cited 2014 Oct 15]. Available from: http://harvardschoolofpublichealth.org/ihsg/publications/pdf/No-19.PDF

69. Ron Levey I, Wang W. Unravelling the quality of HIV counselling and testing services in the private and public sectors in Zambia. Health Policy Plan. 2014;29:i30–7.

70. ACTwatch, Society for Family Health Zambia. Household survey, Zambia, 2011 survey report [Internet]. Population Services International; 2011 [cited 2014 Oct 14]. Available from: http://www.actwatch.info/sites/default/files/content/publications/attachments/ACTwatch%20HH%20Report%20Zambia%202011.pdf

71. Management Sciences for Health. Drug sellers initiatives projects [Internet]. 2013 [cited 2014 Oct 15]. Available from: http://www.drugsellerinitiatives.org/about/projects/

72. Management Sciences for Health. Final report: development and implementation of the ZAAI accreditation program [Internet]. Zambia Ministry of Health; 2011 [cited 2014 Nov 5]. Available from: http://www.drugsellerinitiatives.org/wp-content/uploads/ZAAI-Final-Accreditation-Report-May-26-2011.pdf

73. Friedman J, Fundafunda B, Makumba J, Sjoblom M, Vledder M, Yadav P. Zambia: subsidized ACTs and RDTs in the private sector - preliminary results from the Zambia Access to ACTs Pilot Initiative (ZAAI) [Internet]. World Bank; 2011 [cited 2014 Oct 15]. Available from: http://siteresources.worldbank.org/INTDEVIMPEVAINI/Resources/3998199-1285617002143/7430173-1328726021252/Friedman_Zambia.pdf

74. Rutta E, Kibassa B, McKinnon B, Liana J, Mbwasi R, Mlaki W, et al. Increasing access to subsidized artemisinin-based combination therapy through accredited drug dispensing outlets in Tanzania. Health Res. Policy Syst. 2011;9:22.

75. Management Sciences for Health. Chapter 32: drug seller initiatives. MDS-3 Manag. Access Med. Health Technol. Arlington, VA: Management Sciences for Health; 2012. p. 613–27.

76. Center for Pharmaceutical Management. Accredited drug dispensing outlets in Tanzania: strategies for enhancing access to medicines program final report [Internet]. Management Sciences for Health; 2008. Available from: http://projects.msh.org/news-bureau/upload/SEAM-Tanzania_Final_ADDO.pdf

77. Rutta E. Medicines in health systems: advancing access, affordability and appropriate use. Chapter 5 - annex 1: accredited drug dispensing outlets. [Internet]. World Health Organization; 2014 [cited 2014 Dec 8]. Available from: http://www.who.int/alliance-hpsr/resources/FR_Ch5_Annex1.pdf

78. The Parliament of Zambia. The medicines and allied substances act, 2013 [Internet]. Government of the Republic of Zambia; 2013 [cited 2014 Oct 15]. Available from: www.parliament.gov.zm/index.php?option=com_docman&task=doc_download&gid=1089&Itemid=113

79. Mouzin E, Sedlmayr R, Miller J, Steketee R, Banda P, Chiyota G, et al. Business investing in malaria control: economic returns and a healthy workforce in Africa [Internet]. World Health Organization and Roll Back Malaria Partnership Secretariat; 2011 [cited 2014 Sep 23]. Available from: http://www.path.org/publications/files/MCP_rbm_pi_rpt_6.pdf

80. Chanda E, Kamuliwo M, Steketee RW, Macdonald MB, Babaniyi O, Mukonka VM. An overview of the malaria control programme in Zambia. ISRN Prev. Med. 2013;2013:1–8.

81. Sanders KC, Rundi C, Jelip J, Rashman Y, Smith Gueye C, Gosling RD. Eliminating malaria in Malaysia: the role of partnerships between the public and commercial sectors in Sabah. Malar. J. 2014;13:24.

82. Zambia Ministry of Health. Assessment of the health information system in Zambia [Internet]. 2007 [cited 2014 Oct 15]. Available from: http://www.who.int/healthmetrics/library/countries/HMN_ZMB_Assess_Final_2007_04_en.pdf
